# Supplementary material for: Cost of SARS-CoV-2 self-test distribution programmes by different modalities: a micro-costing study in five countries (Brazil, Georgia, Malaysia, Ethiopia and the Philippines)
Source: BMJ Open. 2024 Apr 17;14(4):e078852. doi: 10.1136/bmjopen-2023-078852 (PMC11029185; doi:10.1136/bmjopen-2023-078852)
Supplement: Supplementary data [file bmjopen-2023-078852supp001.pdf]

Supplementary Materials

Appendices of Cost Categories with Descriptions

Table of Contents

Table A1. *Cost categories with descriptions for Pelotas, Brazil* …..…………………………..... p. 1

Table A2. *Cost categories with descriptions for Afogados da Ingazeira, Brazil* …………….. p. 2

Table A3. *Cost categories with descriptions for Georgia* …………………..………………… p. 3

Table A4. *Cost categories with descriptions for Malaysia* ..………………………………….. p. 5

Table A5. *Cost categories with descriptions for Ethiopia* ..…………………………………... p. 6

Table A6. *Cost categories with descriptions for the Philippines* ..…………………..……….. p. 8

Table A7. *Program characteristics and all-inclusive cost per test* …………………………. p. 10

Table A8. *CHEERS Checklist* ……………………………………………………………….. p. 11

Hansen MA, *et al. BMJ Open* 2024; 14:e078852. doi: 10.1136/bmjopen-2023-078852

Table A1. Cost categories with descriptions for Pelotas, Brazil. For every cost category, all costs included, and any assumptions made are listed. All costs are reported in 2022 USD.

Cost category	Description & explanation
Test procurement	● GeneFinder COVID-19 Ag Self-Test from Osang Healthcare (South Korea)<br>● Self-test unit price (including shipping/importation, taxes, duties, custom clearance): $4.50<br>● 10,000 self-tests procured
Test distribution	● Single shipment from Sao Paulo, Brazil to the workplace storage facility in Pelotas, Brazil at a cost of $0.19 per test.
Test storage	● Tests were stored on-site at the workplace and incurred no additional costs for storage space.<br>● Costs included were employee time required to rearrange storage facility contents to accommodate self-test shipment (2 x staff, 2 hours each x salary per hour).
Training	● Training of pilot beneficiaries on program and proper self-test use.<br>● Training costs were directly related to staff time/salary and are included in staff-management and staff-service delivery cost categories.
Communication	● All communication materials were digital and included posters, banners, and videos to promote COVID-19 self-testing to beneficiaries.<br>● Costs included under communication were for the design of materials (hourly cost of designer and consultant: 80 hours x $40/hour and 60 hours x $20/hour).
Staff: Management	● Project implementation support is a combination of internal employees and external partners.<br>● Four internal workplace employees with partial dedication (between 4-8 hours per month) and one external consultant (allocated 127 hours for the period of the pilot).<br>● Costs include staff-time and salary dedicated to management, oversight and support of the testing program and the program manager.
Staff: Service delivery	● 1 person was hired to be a dedicated full-time program manager of the testing program for the duration of the pilot.<br>● Duties include enrolment, training, test distribution, and data collection/management.
Data and reporting	● Self-test results are reported to the program manager and uploaded to the digital platform.<br>● The cost of the digital platform comprises development costs incurred by IT personnel (80 hours multiplied by staff hourly rate).<br>● Data and reporting costs also include the cost to host meetings/working groups four times over the course of the pilot for 7 people on average at a cost of $57 per person per meeting and $150 per meeting (venue, catering).<br>● Staff time costs that relate activities conducted for data and reporting are included under the staff cost categories.
Consumables and supplies	● Not applicable for this pilot, as testing is performed off-site.
Other	● The pilot was conducted at one site. The pilot took place from September 2022 to February 2023. Costing and data collection was completed in December 2022; therefore, some costs and test results were extrapolated for the remaining 2 months of the pilot.

Hansen MA, *et al. BMJ Open* 2024; 14:e078852. doi: 10.1136/bmjopen-2023-078852

Table A2. Cost categories with descriptions for Afogados da Ingazeira, Brazil. For every cost category, all costs included, and any assumptions made are listed. All costs are reported in 2022 USD.

Cost category	Description & explanation
Test procurement	● COVID-19 self-test kit: Alerta COVID-19 Ag Self-Test manufactured by WAMA Diagnóstica based in Sao Carlos, Brazil<br>● Self-test unit price (including shipping, taxes, duties, custom clearance): $2.00<br>● 26,000 self-tests procured
Test distribution	● Tests were moved from a central storage facility to the Education and Health Secretariat offices using a municipal transport system at no extra cost.<br>● Tests were distributed from Education and Health Secretariat offices to the respective managers of each of the 34 sites included in the pilot study. During the time of the pilot, fuel costs were observed to be $1/liter. Considering the fuel economy for a light duty vehicle in Brazil and maintenance expenses, transportation of 26,000 self-tests to all 34 sites was calculated to be $850.
Test storage	● Tests were stored at an existing storage facility that was centrally located to the Education and Health Secretariats where they were delivered by the manufacturer. No additional cost was incurred from use of the storage space.<br>● 4 palletes to place the boxes holding self-tests were purchased at $37.10 each.
Training	● 2 training workshops were hosted and catered for, with a session providing instructions on how to use the self-test. $400 was budgeted for each training workshop.<br>● A training guide was provided to pilot staff to sensitise staff regarding different self-test procedures as courtesy of the test manufacturer (no cost associated).<br>● 12 hours of initial training hosted by an external consulting agency for a fixed once-off cost of $1656.<br>● Subsequent training sessions on self-test distribution and use were hosted online at no extra cost.
Communication	● Three review meetings were hosted and catered to discuss and communicate the launch of the pilot, progress, and results of the self-test pilot. $400 was budgeted for each review meeting.<br>● Advertisement posters, pamphlets, and stickers were designed and distributed to the beneficiaries at initiation. The communication materials explain when to use tests and what to do depending on the result. The materials cost $220.
Staff: Management	● Project implementation support is provided by two external consulting agencies at a cost of $552 USD per month, as well as a fixed once-off cost of $7,112.
Staff: Service delivery	● 34 individuals comprising four different working groups (operational team, monitoring team, quality control team and management team) contributed approximately 5% of their working time to the project at a cost of $24.29 per individual per month.
Data and reporting	● Self-tests were recorded by beneficiaries in a Google Form that was sent out weekly. No cost was associated with the distribution and design of the Google Form.<br>● Data was managed by two site managers at a rate of $30 per individual per month.
Consumables and supplies	● Not applicable for this pilot, as testing is performed off-site.
Other	● The pilot was conducted at a total of 34 sites. The pilot took place during October 2022 to March 2023. Costing and data collection was completed in December 2022; therefore, some costs and test results were extrapolated for the remaining 3 months of the pilot.

Hansen MA, *et al. BMJ Open* 2024; 14:e078852. doi: 10.1136/bmjopen-2023-078852

Table A3. Cost categories with descriptions for Georgia. For every cost category, all costs included, and any assumptions made are listed. All costs are reported in 2022 USD.

**Cost category**	**Description & explanation**
Test procurement	● COVID-19 self-test kit: On-site COVID-19 Ag Self-Test® (CTK Biotech, California, USA)<br>● Self-test unit price (including shipping, taxes, duties): $2.00<br>● 90,0000 self-tests procured
Test distribution	● Fuel for pilot staff to transport tests from the National Centres for Disease Control (NCDC) to Primary Healthcare Center in Mestia, and from there to schools in the Svaneti region.<br>● Only costs for transportation of tests to schools are accounted for. The NCDC already makes regular trips to the health care facilities and nursing homes for delivery of other supplies through an established delivery system. The additional cost, as well as the opportunity cost of distributing self-tests was considered negligible.
Test storage	● No additional storage space had to be rented to store tests. Every site owned a storage of their own where tests were stored and there was no opportunity cost to the storing of tests.<br>● No additional supplies were purchased to store tests.<br>● Storage of the tests at NCDC was considered negligible.
Training	● A workshop for each of the modalities was provided to train staff (facilitators, principal investigators, project managers) to perform actions needed in the pilot. Costs for these workshops consisted of fuel for transportation, flights (for the school modality), printing supporting materials, supplies (hand sanitizers and masks), venue and hotel costs. - a total of 5 trainings<br>● Allocation of shared training costs for schools, nursing homes and hospital/clinic modalities was based on the number of facilitators allocated to the different modalities.
Communication	● NCDC Communication staff (marketer, social media manager, designer, health promotion, social media coordinator).<br>● Making of videos, social media posts etc. on self-testing and general testing.<br>● Pens, bags, brochures, posters, banners.
Staff: Management	● Salary cost of time spent by:<br>○ Principal investigators (PI and co-PI) at NCDC<br>○ Project managers (PM) at NCDC as well as sites (total of 15)<br>○ Project implementation and support including consultant time<br>○ Finance and admin support from NCDC<br>● Used average Georgian salaries for the current staff position (instead of a stipend paid to staff by FIND to conduct the pilot) to ensure better alignment with the actual costs of scaling up test distribution.<br>● Estimated the average proportion of their time each month that is dedicated to COVID-19 self-test implementation and operations.<br>● Pilot-related staff, such as PMs included as time largely spent on implementation (with a small component spent on research-specific activities).<br>● Included 2 months preparation time for the pilot.
Staff: service delivery	● This includes the salary of the facilitators at each of the sites (NCDC facilitators (9), hospitals (24), clinics (7), nursing homes (4), and schools (24), and then the average proportion of their time each month that is dedicated to COVID-19 self-test operations.<br>● Facilitator activities included:<br>○ Facilitator time spent on collecting COVID-19 self-tests to bring to appropriate testing facility (i.e., workplace, schools)<br>○ Facilitator time spent on storing and organizing COVID-19 self-tests<br>○ Facilitator time spent on distributing the COVID-19 self-test to participants<br>○ Facilitator time spent on training clients/participants on how to use the COVID-19 self-test, including information and sensitization activities

	• Used average Georgian salaries for the current staff position (instead of a stipend paid to staff by FIND to conduct the pilot) to ensure better alignment with the actual costs of scaling up test distribution.
Data and reporting	• Costs associated with the development and use of a database/app where results are documented and reported.<br>• Staff time of data entry operators at hospitals (6), clinics (2) and nursing homes (2). (Facilitators conduct data entry at schools). Data entry operators are responsible for record keeping after COVID-19 self-tests are distributed, maintaining registers, data compilation and entry. Beneficiaries test weekly and, if positive, call in immediately for the data operator to record it. If negative, the beneficiary will submit the result at work.<br>• Technical Working Group meetings to report and share results (venue, food, IT, and transportation) – a total of 3.<br>• Data support from the NCDC in terms of staff time (5).

Table A4. Cost categories with descriptions for Malaysia. For every cost category, all costs included, and any assumptions made are listed. All costs are reported in 2022 USD.

Cost category	Description & explanation
Test procurement	• COVID-19 self-test kit: FlowFlex Ag-RDTs manufactured by ACON Biotech (Hangzhou) Co., Ltd.<br>• Self-test unit price (including shipping, taxes, duties, custom clearance): $1.55<br>• 45,000 self-tests procured
Test distribution	• Tests were shipped from the central storage facility to each site through a third-party courier at a rate of $0.0121 per test. This cost included door-to-door delivery, driver salary, fuel, vehicle, etc.<br>• For the 45,000 tests procured for the pilot, test distribution costs totalled $545.
Test storage	• Tests were stored in a central storage facility in Kuala Lumpur at a cost of $420 per month.<br>• A temperature data logger was purchased to ensure temperature regulation of the storage facility as a once off cost of $20.
Training	• 2 in-person training sessions were hosted for a research assistant dedicated to the self-testing pilot. Travel associated with attending each session totalled $286.<br>• 1 training session was hosted at each site (4 total) to train site managers on the self-testing pilot. $915 was incurred for each training session per site.<br>• A sensitization session was hosted at each site (4 total) to train beneficiaries on how to use the FlowFlex Ag-RDT. $458 was incurred for each training session per site.
Communication	• Supplementary information and reference guides for the self-tests were provided to each beneficiary. $1030 was budgeted for these communication materials.<br>• 3 working group meetings were hosted to monitor and communicate upon the reception and progress of the COVID-19 self-testing pilot. $1145 was budgeted for each working group meeting.
Staff: Management	• Project implementation support is provided by an external organisation for $1438 per month.
Staff: Service delivery	• A research assistant was dedicated to delivering the self-tests to their respective sites and hosting the sensitization sessions for the beneficiaries. Salary and travel expenses for this position resulted in a total cost of $1145 per month.
Data and reporting	• Self-tests were recorded by beneficiaries in a Google Form that was sent out weekly and managed by the project's dedicated research assistant. No additional cost was associated with the distribution and management of the Google Form.<br>• A sim card was purchased to follow-up with beneficiaries via phone who did not report their test results on the Google form for $11 per month.
Consumables and supplies	• Not applicable for this pilot, as testing is performed off-site.
Other	• The pilot was conducted at a total of 2 sites. The pilot took place during November 2022 to April 2023. Costing and data collection was completed in December 2022; therefore, some costs and test results were extrapolated for the remaining 2 months of the pilot.

Hansen MA, *et al. BMJ Open* 2024; 14:e078852. doi: 10.1136/bmjopen-2023-078852

Table A5. Cost categories with descriptions for Ethiopia. For every cost category, all costs included, and any assumptions made are listed. All costs are reported in 2022 USD.

**Cost category**	**Description & explanation**
Test procurement	● COVID-19 self-test kit: Flowflex Acon Biotech self-test kit, ACON Biotech (Hangzhou) Co.,Ltd.<br>● Self-test unit price: $1.00<br>● 1,250 self-tests procured
Test distribution	● Transportation cost from the airport to health care facilities. Once-off cost ($140) for all imported tests used to estimate a cost per self-test kit transported.
Test storage	● No additional storage space had to be rented to store tests. Every site owned a storage of their own where tests were stored and there was no opportunity cost to the storing of tests.<br>● No additional supplies were purchased to store tests.
Training	● A video was produced and procured for self-test kit demonstration purposes. Field coordinators initially watched the video themselves (as part of training). The video was also shown to patients to demonstrate proper self-test kit usage.<br>● Once-off cost of $500 for video production.
Communication	● Mobile card usage for four field coordinators (2 per site) to provide updates on the study.<br>● Monthly cost per mobile card: $14.81
Staff: Management	● Salary cost of time spent by:<br>○ Cleaner, administrative assistant, monitoring & evaluation officer, and program coordinator at sites (total of 8 sites)<br>○ Project implementation and support including consultant time from implementing partner<br>○ Finance and administration support from implementing partner<br>● Used average Ethiopian salaries for the current staff position (instead of a salary paid by the implementing partner to conduct the pilot) to ensure better alignment with the actual costs of scaling up test distribution.<br>● Estimated the average proportion of their time each month that is dedicated to COVID-19 self-test implementation and operations.<br>● Allocated the time of all staff to the 2 sites used in the costing analysis based on test-kit distribution For the routine implementation scenario analysis, the level of effort of the monitoring and evaluation officer was reduced, and the salary cost of the monitoring and evaluation officer and programme coordinator was aligned with that of the staff cadre that would likely fill these positions should the programme be scaled.
Staff: Service delivery	● This includes the salary of the OPD physicians (1 per site), nurses and field coordinators (2 per site) at each of the sites (2 sites).<br>● Staff: service delivery activities included:<br>○ OPD physicians: time spent on evaluation clients if they are eligible for self-testing and linking them to field coordinators (1 hour per day)<br>○ Nurses: time spent on observing clients execute self-testing, collect, and process the sample and finally read the result<br>○ Field coordinators: time spent on arrival and registration of client, showing client a demonstration video, pre-test counselling, preparing the testing area and conclude by providing post-test counselling/information<br>● The direct staff cost for nurses and field coordinators was estimated on a per test basis by observing time estimates spent on service delivery and salary cost per minute: staff nurse (25 minutes per client); field coordinator (42 minutes per client).

	• Used average Ethiopian salaries for the staff cadre required to perform the task (instead of a salary paid to staff by the implementing partner to conduct the pilot) to ensure better alignment with the actual costs of scaling up test distribution.
Data and reporting	• Field coordinators provide post-counselling/information to clients and document and report the self-test result.<br>• Unlimited internet SIM card procured for four field coordinators (acting as data collectors; 2 per site) for facilitation of data entry on REDCap: $16.67 per month.
Consumables and supplies	• Costed hand sanitizer, gloves, and alcohol for disinfecting the testing area on a per test basis.<br>• Costed masks and biohazard bags used by the facility and staff to oversee the self-testing.
Other	• The pilot was conducted at a total of 8 sites. However, given costing timelines, costs were only collected for 2 sites. These sites are however representative of the other sites.<br>• Costs for the video production and overhead staff management were allocated to the 2 sites based on test-kit distribution (46% of test kits were distributed at the 2 sites that were costed).

Hansen MA, *et al. BMJ Open* 2024; 14:e078852. doi: 10.1136/bmjopen-2023-078852

Table A6. Cost categories with descriptions for the Philippines. For every cost category, all costs included, and any assumptions made are listed. All costs are reported in 2022 USD.

Cost category	Description & explanation
Test procurement	• COVID-19 self-test kit: Flowflex Acon Biotech self-test kit, ACON Biotech (Hangzhou) Co.,Ltd.<br>• Self-test unit price: $1.32 (including shipment of test from the local distributor – taxes and duties)<br>• 2,000 self-tests procured
Test distribution	• Transport billed in bulk inclusive of porter's/driver's salary, vehicle rental, and applicable fuel, toll fees, and taxes. Fixed cost per day, per site, per month.<br>• Tests are distributed directly to the partner sites.
Test storage	• No additional storage space had to be rented to store tests at the central office. Store to capacity and re-order when need arises.<br>• Each site has storage of its own and no opportunity cost to the storing of tests.
Training	• A 1-day training workshop was conducted with all partner site staff (50 in total).<br>• Each participant received 2 demonstration tests.<br>• A cost per person trained per day was calculated to estimate the cost of training the staff at one site.
Communication	• All site staff receive a monthly communication allowance inclusive of phone, internet service provider subsidy and data transfer to servers.<br>• Management staff also receive a monthly communication allowance to manage all 7 sites. This cost is allocated to the costed site using 1/7 factor.<br>• Printing papers, stationery, cartridges for brochures, Information Education Communication (IEC) materials, and posters, banners, and brochures – once-off per site.
Staff: Management	• 1 x Project implementer Research officer (supporting all sites).<br>• 1x Project M&E Officer – solely contracted by project implementer to supervise across all sites.<br>• 1 x Site Supervisor (clinic staff) at nurse salary grade 19 (government salary grade)<br>• Estimated the average proportion of their time each month that is dedicated to COVID-19 self-test implementation and operations:<br>○ 5 hours per 8-hour day for the Project implementer Research officer<br>○ 5 hours per 8-hour day for the Project M&E Research officer<br>○ 1 hour per 8-hour day for the Site Supervisor (clinic staff)<br>• Research staff time spent on self-test distribution allocated per site (monthly salary/7).<br>• M&E Research staff time spent on self-test distribution allocated per site (monthly salary/7).<br>• Included 1-month of preparation time.
Staff: Service delivery	• 1x Test counsellor –at medical technologist II salary grade 15 (government salary grade).<br>• 1x Test administrator – at Nurse Salary I with salary grade 15.<br>• Estimated the average proportion of their time each month that is dedicated to COVID-19 self-test implementation and operations (3 hours per 8-hour day).
Data and Reporting	• 1x Site Data Entry Operator – Project implementer assigned staff on-site, Nursing Attendant II w/ Salary Grade 6 (government salary grade).<br>• Estimated the average proportion of their time each month that is dedicated to COVID-19 self-test implementation and operations:<br>○ 100% of their time for the Data Entry Operator

Consumables and Supplies	• Hygiene essentials: infection control kits available on-site as part of IPC procedures (alcohol, masks, gloves, hand soap, paracetamol, disinfectants, thermometer).<br>• Office supplies for staff: documentation and office essentials (printing papers, stationery, cartridges, ball pens).
Other	• The pilot was conducted at a total of 7 sites. Only 1 site was costed. This site was considered typical of the other sites; set-up is the same across sites, only the scale of testing differs.<br>• Costs for the staff that oversee operations across sites, their communication allowance and the supplies and consumables were allocated to one site based on the factor of 1/7.

Hansen MA, *et al. BMJ Open* 2024; 14:e078852. doi: 10.1136/bmjopen-2023-078852

Table A7. Program characteristics and all-inclusive cost per test. These characteristics and costs are reported for each country during the pilot (Scenario 1) and annual routine periods of implementation (Scenario 2 and 3). This is an extended version of Table 4 in the manuscript.

			**Scenario 1 – pilot implementation**			**Scenario 2 – routine implementation**			**Scenario 3 – scaled peak implementation**			
**Country**	**Testing location**	**Provider assisted or unassisted**	**# tests distributed**	Purchase price of self-test kit	**Cost per test:** all-inclusive	**# tests distributed**	**Cost per test:** using current self-test kit price	**Cost per test:** using reduced self-test kit price	**# tests distributed**	**Cost per test:** using current self-test kit price	**Cost per test:** using reduced self-test kit price	**Cost per test:** with integrated staff, data, & reporting
**Brazil (Pelotas)**	Primarily off-site	Unassisted	**10,000**	$4.50	$7.38	**20,000**	$6.54	$4.29	**40,000**	$5.62	$3.37	$2.55
**Brazil (Afogados)**	Primarily off-site	Unassisted	**26,000**	$2.00	$2.82	**52,000**	$2.59	$1.59	**104,000**	$2.30	$1.30	$1.05
**Georgia**	Off-site	Unassisted	**90,000**	$2.00	$4.44	**300,000**	$3.34	$2.34	**600,000**	$2.95	$1.95	$1.61
**Malaysia**	Off-site	Unassisted	**45,000**	$1.55	$2.44	**135,000**	$2.06	$1.28	**270,000**	$1.82	$1.04	$0.86
**Ethiopia**	On-site	Provider assisted	**158**	$1.00	$12.78	**5,040**	$4.22	$3.72	**10,080**	$3.80	$3.30	$1.97
**Philippines**	On-Site	Unassisted	**2,000**	$1.32	$4.99	**6,000**	$4.51	$3.85	**12,000**	$2.92	$2.26	$0.95

Table A8. CHEERS Checklist

Topic	No.	Item	Location where item is reported
**Title**			
	1	Identify the study as an economic evaluation and specify the interventions being compared.	Title
**Abstract**			
	2	Provide a structured summary that highlights context, key methods, results, and alternative analyses.	Abstract
**Introduction**			
**Background and objectives**	3	Give the context for the study, the study question, and its practical relevance for decision making in policy or practice.	Introduction, starting at line 74
**Methods**			
**Health economic analysis plan**	4	Indicate whether a health economic analysis plan was developed and where available.	Methods, starting at line 129 and 147
**Study population**	5	Describe characteristics of the study population (such as age range, demographics, socioeconomic, or clinical characteristics).	Methods, start at line 129
**Setting and location**	6	Provide relevant contextual information that may influence findings.	Methods, start at line 140
**Comparators**	7	Describe the interventions or strategies being compared and why chosen.	Methods, start at line 172
**Perspective**	8	State the perspective(s) adopted by the study and why chosen.	Methods, line 148
**Time horizon**	9	State the time horizon for the study and why appropriate.	Methods, line 151
**Discount rate**	10	Report the discount rate(s) and reason chosen.	Not applicable
**Selection of outcomes**	11	Describe what outcomes were used as the measure(s) of benefit(s) and harm(s).	Not applicable
**Measurement of outcomes**	12	Describe how outcomes used to capture benefit(s) and harm(s) were measured.	Not applicable
**Valuation of outcomes**	13	Describe the population and methods used to measure and value outcomes.	Not applicable
**Measurement and valuation of resources and costs**	14	Describe how costs were valued.	Methods, line 147
**Currency, price date, and conversion**	15	Report the dates of the estimated resource quantities and unit costs, plus the currency and year of conversion.	Methods, line 150

Hansen MA, *et al. BMJ Open* 2024; 14:e078852. doi: 10.1136/bmjopen-2023-078852

Topic	No.	Item	Location where item is reported
**Rationale and description of model**	16	If modelling is used, describe in detail and why used. Report if the model is publicly available and where it can be accessed.	Not applicable
**Analytics and assumptions**	17	Describe any methods for analysing or statistically transforming data, any extrapolation methods, and approaches for validating any model used.	Methods, line 172
**Characterising heterogeneity**	18	Describe any methods used for estimating how the results of the study vary for subgroups.	Methods, starting at line 172
**Characterising distributional effects**	19	Describe how impacts are distributed across different individuals or adjustments made to reflect priority populations.	Methods, starting at line 172
**Characterising uncertainty**	20	Describe methods to characterise any sources of uncertainty in the analysis.	Methods, starting at line 172
**Approach to engagement with patients and others affected by the study**	21	Describe any approaches to engage patients or service recipients, the general public, communities, or stakeholders (such as clinicians or payers) in the design of the study.	See appendices
**Results**			
**Study parameters**	22	Report all analytic inputs (such as values, ranges, references) including uncertainty or distributional assumptions.	Not applicable
**Summary of main results**	23	Report the mean values for the main categories of costs and outcomes of interest and summarise them in the most appropriate overall measure.	Results, Figure 1
**Effect of uncertainty**	24	Describe how uncertainty about analytic judgments, inputs, or projections affect findings. Report the effect of choice of discount rate and time horizon, if applicable.	Not applicable
**Effect of engagement with patients and others affected by the study**	25	Report on any difference patient/service recipient, general public, community, or stakeholder involvement made to the approach or findings of the study	Results, starting at line 212
**Discussion**			
**Study findings, limitations, generalisability, and current knowledge**	26	Report key findings, limitations, ethical or equity considerations not captured, and how these could affect patients, policy, or practice.	Discussion, starting at line 254
**Other relevant information**			
**Source of funding**	27	Describe how the study was funded and any role of the funder in the identification, design, conduct, and reporting of the analysis	Line 442
**Conflicts of interest**	28	Report authors conflicts of interest according to journal or International Committee of Medical Journal Editors requirements.	Line 437

Hansen MA, *et al. BMJ Open* 2024; 14:e078852. doi: 10.1136/bmjopen-2023-078852
