# Supplementary material for: Cost of SARS-CoV-2 self-test distribution programmes by different modalities: a micro-costing study in five countries (Brazil, Georgia, Malaysia, Ethiopia and the Philippines)
Source: BMJ Open. 2024 Apr 17;14(4):e078852. doi: 10.1136/bmjopen-2023-078852 (PMC11029185; doi:10.1136/bmjopen-2023-078852)
Supplement: Author's manuscript [file bmjopen-2023-078852.draft_revisions.pdf]

BMJ Open is committed to open peer review. As part of this commitment we make the peer review history of every article we publish publicly available.

When an article is published we post the peer reviewers' comments and the authors' responses online. We also post the versions of the paper that were used during peer review. These are the versions that the peer review comments apply to.

The versions of the paper that follow are the versions that were submitted during the peer review process. They are not the versions of record or the final published versions. They should not be cited or distributed as the published version of this manuscript.

BMJ Open is an open access journal and the full, final, typeset and author-corrected version of record of the manuscript is available on our site with no access controls, subscription charges or pay-per-view fees (http://bmjopen.bmj.com).

If you have any questions on BMJ Open's open peer review process please email info.bmjopen@bmj.com

BMJ Open

The cost of SARS-CoV-2 self-test distribution programs by different modalities in five countries

Journal:	*BMJ Open*
Manuscript ID	bmjopen-2023-078852
Article Type:	Original research
Date Submitted by the Author:	14-Aug-2023
Complete List of Authors:	Hansen, Megan; Amsterdam UMC Location AMC, Medical Microbiology; Boston University School of Public Health, Global Health<br>Lekodeba, Nkgomeleng; Health Economics and Epidemiology Research Office<br>Chevalier, Joshua; Amsterdam UMC Locatie AMC, Medical Microbiology; Boston University School of Public Health, Global Health<br>Ockhuisen, Tom; Amsterdam UMC Location AMC, Medical Microbiology<br>del Rey-Puech, Paula; Foundation for Innovative New Diagnostics<br>Marban-Castro, Elena; Foundation for Innovative New Diagnostics<br>Martínez-Pérez, Guillermo; Foundation for Innovative New Diagnostics<br>Shilton, Sonjelle; Foundation for Innovative New Diagnostics<br>Radzi Abu Hassan, Muhammad; Hospital Sultanah Bahiyah, Clinical Research Center<br>Getia, Vladimir; National Center for Disease Control and Public Health, Lugar Research Center<br>Weinert-Mizuschima, Catiuscia; LIFEMED<br>Tenorio Bezerra, Maria Isabelle; Secretaria Municipal de Saúde<br>Chala, Lensa; KNCV Tuberculosis Foundation<br>Leong, Robert; KNCV Tuberculosis Foundation<br>Peregino, Remilekun; The Aurum Institute for Health Research<br>Keller, Sara; KNCV Tuberculosis Foundation<br>Spruijt, Ineke; KNCV Tuberculosis Foundation<br>Johnson, Cheryl; World Health Organization<br>Girdwood, Sarah; Foundation for Innovative New Diagnostics; Health Economics and Epidemiology Research Office<br>Nichols, Brooke E.; Amsterdam UMC Location AMC, Medical Microbiology; Foundation for Innovative New Diagnostics
Keywords:	COVID-19, Health economics < HEALTH SERVICES ADMINISTRATION & MANAGEMENT, Public health < INFECTIOUS DISEASES, Respiratory infections < THORACIC MEDICINE

SCHOLARONE™
Manuscripts

I, the Submitting Author has the right to grant and does grant on behalf of all authors of the Work (as defined in the below author licence), an exclusive licence and/or a non-exclusive licence for contributions from authors who are: i) UK Crown employees; ii) where BMJ has agreed a CC-BY licence shall apply, and/or iii) in accordance with the terms applicable for US Federal Government officers or employees acting as part of their official duties; on a worldwide, perpetual, irrevocable, royalty-free basis to BMJ Publishing Group Ltd ("BMJ") its licensees and where the relevant Journal is co-owned by BMJ to the co-owners of the Journal, to publish the Work in this journal and any other BMJ products and to exploit all rights, as set out in our licence.

The Submitting Author accepts and understands that any supply made under these terms is made by BMJ to the Submitting Author unless you are acting as an employee on behalf of your employer or a postgraduate student of an affiliated institution which is paying any applicable article publishing charge ("APC") for Open Access articles. Where the Submitting Author wishes to make the Work available on an Open Access basis (and intends to pay the relevant APC), the terms of reuse of such Open Access shall be governed by a Creative Commons licence – details of these licences and which Creative Commons licence will apply to this Work are set out in our licence referred to above.

Other than as permitted in any relevant BMJ Author's Self Archiving Policies, I confirm this Work has not been accepted for publication elsewhere, is not being considered for publication elsewhere and does not duplicate material already published. I confirm all authors consent to publication of this Work and authorise the granting of this licence.

For peer review only

The cost of SARS-CoV-2 self-test distribution programs by different modalities in five countries

Authors: Megan A. Hansen[1,2], Nkgomeleng Lekodeba[3], Joshua M. Chevalier[1,2], Tom Ockhuisen[1], Paula del Rey-Puech[4], Elena Marban-Castro[4], Guillermo Zohar Martinez-Perez[4], Sonjelle Shilton[4], Muhammad Radzi Abu Hassan[5], Vladimir Getia[6], Catiuscia Weinert-Mizuschima[7], Maria Isabelle Tenorio Bezerra[8], Lensa Chala[9], Robert Leong[10], Remilekun Roland Peregino[11], Sara Keller[12], Ineke Spruijt[12], Cheryl Johnson[13], Sarah J. Girdwood[3,4*], Brooke E. Nichols[1,3,4*]

Affiliations:

1. Department of Medical Microbiology, Amsterdam University Medical Center, Amsterdam, The Netherlands
2. Department of Global Health, Boston University School of Public Health, Boston, Massachusetts, United States of America
3. Health Economics and Epidemiology Research Office, Faculty of Health Sciences, University of the Witwatersrand, Johannesburg, South Africa
4. Foundation for Innovative New Diagnostics (FIND), The Global Alliance for Diagnostics, Geneva, Switzerland
5. Clinical Research Center, Hospital Sultanah Bahiyah, Alor Setar, Malaysia
6. Lugar Research Center, CNational Centers for Disease Control and Public Health, Tbilisi, Georgia
7. LIFEMED, Pelotas, Rio Grande do Sul, Brazil
8. Secretaria Municipal de Saúde, Afogados da Ingazeira, Pernambuco, Brazil
9. KNCV Tuberculosis Foundation, Addis Ababa, Ethiopia
10. KNCV Tuberculosis Foundation, Manilla, The Philippines
11. Aurum Institute, Accra, Ghana
12. KNCV Tuberculosis Foundation, The Hague, The Netherlands
13. World Health Organization (WHO), Geneva, Switzerland

Correspondence: brooke.nichols@finddx.org m.hansen@amsterdam.nl *shared contribution

For peer review only - http://bmjopen.bmj.com/site/about/guidelines.xhtml

Abstract

Introduction:

Diagnostic testing is an important tool to combat the COVID-19 pandemic, yet access to and uptake of testing varies widely three years into the pandemic. The World Health Organization recommends the use of COVID-19 self-testing as an option to help expand testing access. We aimed to calculate the cost of providing COVID-19 self-testing across countries and distribution modalities.

Methods:

We estimated economic costs from the provider-perspective using data collected between August and December 2022 in Brazil, Georgia, Malaysia, Ethiopia, and the Philippines from pilot implementation studies designed to provide COVID-19 self-tests in a variety of settings – namely workplace and healthcare facilities. We calculated the total cost and the cost per self-test kit distributed for three scenarios that differed by costing period (pilot, annual), the number of tests distributed (actual, planned, scaled assuming an epidemic peak), and self-test kit costs (pilot purchase price, 50% reduction).

Results:

Across all five countries, 173,000 kits were distributed during pilot implementation with the cost/test distributed ranging from $2.44–$12.78. The cost/self-test kit distributed was lowest in the scenario that assumed implementation over a longer period (year), with higher test demand (peak), and a test-kit price reduction of 50% ($1.04–$3.07). Across all countries and scenarios, test procurement occupied the greatest proportion of costs: 58%–87% for countries with off-site self-testing (outside the workplace, e.g., home), 15%–50% for countries with on-site self-testing (at the workplace). Staffing was the next key cost-driver, particularly for distribution modalities that had on-site self-testing (29%–35%) versus off-site self-testing (7%–27%).

Conclusions:

Our results indicate that it is likely to cost between $2.44–$12.78 per test to distribute COVID-19 self-tests across common settings in five heterogeneous countries. Cost-effectiveness analyses using these results will allow policymakers to make informed decisions on optimally scaling-up COVID-19 self-test distribution programs across diverse settings and evolving needs.

For peer review only - http://bmjopen.bmj.com/site/about/guidelines.xhtml

Introduction

As of July 2023, the COVID-19 pandemic has led to nearly 768 million cases and 6.9 million deaths worldwide [1]. While testing has been acknowledged as a critical tool to combat the spread of SARS-CoV-2, access to testing remains varied nearly three years into the pandemic. In the early stages of the COVID-19 pandemic, polymerase chain reaction (PCR) tests were the preferred method for the diagnosis of SARS-CoV-2. However, as more cases emerged, and transmission became widespread, low-cost ($1.50 per test) antigen-based rapid diagnostic tests have increasingly been utilized to expand access to and uptake of testing [2].

Despite the benefits, many programs found limiting the use of Ag-RDTs to professional administration and use still missed many people in need of testing services [3]. The use of self-testing for Ag-RDTs, where individuals perform and interpret their own diagnostic test, is a mechanism to relieve this human resource constraint and rapidly expand potential testing capacity. These more affordable self-tests also allow individuals to test themselves, promptly learn their results, and take actions that could improve individual health outcomes and/or prevent onward transmission. For these reasons, COVID-19 self-tests have become increasingly popular in high-income countries (HICs) and were recommended by the World Health Organization (WHO) in 2022 [1].

Distribution of self-test kits would allow for the scale-up of COVID-19 testing in limited-resource settings, where regular screening through provider-initiated and/or laboratory-based platforms, such as PCR tests, is constrained. While there are some studies on the use of Ag-RDT self-tests in schools, workplaces, and within the broader community, these studies focus primarily on the usability or accuracy in comparison to provider-offered testing [4–7]. There is limited research on the relative programmatic costs for the implementation and distribution of COVID-19 self-tests through different modalities, especially in low-resource settings or among communities with restricted access to conventional COVID-19 testing, where self-testing may be of most benefit [8].

Most of the empirical evidence on the cost of self-testing programs originates from the Human Immunodeficiency Virus (HIV) self-testing literature [6,9,10], and more recently from Hepatitis C Virus (HCV) self-testing [11]. Studies have demonstrated the relative cost of different methods for rollout of HIV self-testing and found, broadly, that there are multiple ways of successfully distributing HIV self-testing, depending on the budget and desired outcome. For HIV self-testing, expansion of testing beyond primary health care clinics yielded a net increase in life years saved across all distribution models [9]. Workplace

For peer review only - http://bmjopen.bmj.com/site/about/guidelines.xhtml

testing was found to be a very cost-effective modality for HIV self-test distribution. It may be plausible that self-testing for COVID-19 is similarly cost-effective in the workplace.

The HIV literature gives us insights into relative differences in cost of implementation of self-test distribution modalities, as well as which modalities are acceptable, appropriate, and perceived to be convenient for a variety of populations. However, there are differences in modalities and frequency of testing required for HIV or HCV compared to a respiratory virus such as COVID-19. Given these key differences, more evidence is needed to understand the costs related to delivering COVID-19 self-testing through different modalities for future planning – for potential future COVID-19 waves, for other seasonal viruses such as influenza, and possibly for future outbreaks caused by emerging diseases.

Despite the recently popular wide use of COVID-19 self-testing in countries with available public health resources, there has been a paucity of data generated to understand the cost of provision, particularly in lower middle-income countries (LMICs) and middle-income countries (MICs). We aimed to determine the cost per COVID-19 self-test allocated by distribution modality as well as the cost per COVID-19 self-test performed. The overarching aim was to provide costing evidence to governments making decisions regarding the adoption and scale-up of self-test distribution programs to monitor and effectively improve case detection of COVID-19.

Methods

Overview

COVID-19 self-testing pilot studies were implemented in three upper middle-income countries (UMICs): Brazil, Georgia, and Malaysia, as well as one LMIC (the Philippines) and one low-income country (LIC) (Ethiopia). These pilot implementation studies were designed to provide COVID-19 self-tests to employees, their household members, and clinic clients in a variety of different settings – ranging from healthcare settings, to workplaces, to schools (Table 1). The goal of the pilot studies was to assess and continuously improve COVID-19 self-testing distribution strategies, the feasibility of the implementation, and participants' acceptability – as an appropriate intervention to increase uptake of testing and detection of cases of COVID-19 in the target populations [7]. Additionally, these pilot studies aimed to calculate the cost of providing COVID-19 self-testing via several distribution modalities in different countries.

For peer review only - http://bmjopen.bmj.com/site/about/guidelines.xhtml
For peer review only

Study setting and scope

This is a multi-site, prospective cohort study across five countries and multiple workplaces and health facilities (Table 1).

Table 1. Description of distribution models. *Off-site testing: refers to self-testing outside the workplace (e.g., at home) On-site testing: refers to self-testing at the workplace.

Country	Distribution Modality*	Pilot Implementation Time Period	Potential participants and channel	Secondary distribution to household members	Description
Brazil (Pelotas, Rio Grande do Sul)	Staff at a workplace of a private medical supply factory for primarily off-site testing	August 2022 – February 2023	Primary distribution to 358 employees	Yes	For the first month, four self-tests were distributed per participant to perform weekly testing. Afterwards, up to six self-test kits were distributed to staff members per month, with recommended use of one per week or at the onset of any COVID-19 symptoms. Results were recorded through a digital platform used at the workplace (mobile application) and participants were expected to seek confirmatory testing, following national guidelines.
Brazil (Afogados da Ingazeira, Pernambuco)	Professionals at sites in both the public healthcare sector (20) and the public	October 2022 – March 2023	Primary distribution to 344 employees working in healthcare	Yes	During the first month employees were encouraged to perform one self-test per week regardless of if COVID-19 symptoms were present or not (resulting in approximately four tests per month), and an

For peer review only - http://bmjopen.bmj.com/site/about/guidelines.xhtml

	education sector (14) for primarily off-site testing		and 855 employees working in the education sector (up to 1,200 total participants)		additional self-test is recommended if symptoms arise throughout the week. Afterwards, employees were encouraged to self-test in case of symptoms or contact with a positive case. Results were recorded through a survey link sent via Google Forms and participants were expected to seek confirmatory testing, following national guidelines.
Georgia (Tbilisi, Kutaisi and Svaneti region)	Staff at schools in a remote mountainous region (24), nursing homes (2) and healthcare centers (hospitals (2) and clinics (1)) for primarily off-site testing	June 2022 – December 2022	Primary distribution to 2,066 employees	Yes	Primary self-test kit distribution to staff members at pilot sites for weekly testing, as part of the mandatory national testing program, and testing in case of symptoms or contact with a positive case, as well as secondary self-test kit distribution to staff members' household members for use in the case of developing symptoms or having a close contact with a positive COVID-19 case. Results were reported to designated staff right after use to be entered in the national database. Positive staff members were linked to care.
Malaysia (Alor Setar, Sungai	Staff of private manufacturing companies (4)	November 2022 to April 2023	Primary distribution to up to 2,000	Yes	Initial distribution of two self-test kits per staff and household member for self-testing in case of symptomatic/contract/other

For peer review only - http://bmjopen.bmj.com/site/about/guidelines.xhtml

For peer review only

Petani, and wider state of Kedah)	for primarily off-site self-testing		employees		circumstances, following national guidelines. Kits were refilled on an as-needed basis. Participants received training and information materials on-site prior to test distribution. Participants were asked to report test-use and results by Google Form.
Ethiopia (Addis Ababa and regional states of Amhara and Oromia)	Clients at clinics for on-site self-testing (2)	September 2022 – November 2022	Primary distribution to clients eligible for self-testing when visiting the clinics (total tests available 1250)	No	Clients arrived at clinic to seek health care and were screened for eligibility for partaking in the pilot. If the client was eligible, a self-test kit was provided for on-site self-testing. Clients performed test, interpret, and read results while supervised by nurses. If the self-test was positive, the client was sent for confirmatory testing.
Philippines (Biñan, Los Baños, and wider province of Laguna)	Staff at workplace clinics for on-site testing (7)	September to December 2022	Primary distribution to workers working at partner clinics. (total tests available 500/site/month)	No	Distribution of self-test kits at partner workplace, with on-site assigned facility to be its clinic. Distribution agents demonstrated self-test kit use to groups. Participants were tested on-site by a provider under supervision of clinic staff.

Costing inputs

We estimated economic costs from the provider perspective using data collected between August and

For peer review only

For peer review only - http://bmjopen.bmj.com/site/about/guidelines.xhtml

November 2022 in Brazil, Georgia, Malaysia, Ethiopia, and the Philippines. All the costs were either collected in United States Dollars (USD) or the countries' local currency and converted to 2022 USD using the average exchange rate for the period January to October 2022 sourced from the country's central/national banks [12–17].

The costing approach used was a combination of expenditure analysis in estimating financial costs and bottom-up costing to identify any items not included in pilot expenditure records [18]. Cost data was collected across all countries and modalities using a standardized Excel data collection tool that categorized the costs as follows: training, test procurement, distribution and storage, staff costs (management and service delivery), data reporting, supplies and consumables, and communication (Table 2; details for each country and modality in Appendix). Costs in Brazil, Georgia, and Malaysia were collected by while our team travelled to the sites and investigated financial records. Costs in Ethiopia and the Philippines were reported by our in-country research partners.

Shared costs were allocated to each distribution modality using the proportion of tests distributed and the proportion of staff for costs driven by this (for example, training costs). Staff salaries were obtained from the in-country implementing partners and allocated by the proportion of time spent on COVID-19 self-test distribution as estimated by in-country implementing partners. Where appropriate, staff salaries for the equivalent staff cadre within the Ministry of Health or government were used in order to better align with routine implementation assumptions in the future.

Table 2. Cost categories and description

**Cost category**	**Description & explanation**
Test procurement	• Cost of COVID-19 self-test kit unit<br>• Shipping, customs clearance, duties, taxes
Test distribution	• Transportation cost (e.g., fuel) for the transportation of COVID-19 self-tests to sites (including transport to central storage location if relevant)
Test storage	• Purchase or rental of storage space for COVID-19 self-test, such as a warehouse or storage unit<br>• Purchase of any necessary accompanying supplies used for storage (e.g., shelves and boxes)

For peer review only - http://bmjopen.bmj.com/site/about/guidelines.xhtml

Training	• Training sessions of staff that are members of the team that are dedicated to the COVID-19 self-test activities<br>• Training sessions for the participants on how and when to self-test, how to report the results, and where to find more information about self-testing<br>• Design and printing of training materials<br>• Venue, catering, supplies, and transport costs associated with training
Communication	• Designing and ordering advertisement posters and stickers<br>• Designing and printing information materials, such as letters and pamphlets<br>• Designing and distributing digital communication materials (e.g., instructional videos, digital infographics)<br>• Any additional communication activities that may accrue cost, such as awareness trainings<br>• Any internet or mobile associated communication costs
Staff: management	• Cost of staff time spent on activities to manage the COVID-19 self-testing campaign<br>• Cost of time spent on project implementation and support (including any consultant's time where appropriate)
Staff: service delivery	• This includes the salary of the staff, and the average proportion of their time spent dedicated to COVID-19 self-test operations:<br>• Activities include staff time spent on:<br>○ Collecting COVID-19 self-tests to bring to appropriate testing facility (e.g., workplace, clinic)<br>○ Storing and organizing COVID-19 self-tests<br>○ Enrollment sessions for initiation of COVID-19 self-test distribution to participants<br>○ Assisting or administering the COVID-19 self-test to participants<br>○ Training participants on how to use the COVID-19 self-test, including information and sensitization activities, answering questions during the pilot
Data and reporting	• Development and use of a platform where results are documented and reported

For peer review only

For peer review only - http://bmjopen.bmj.com/site/about/guidelines.xhtml

	• Record keeping after COVID-19 self-tests are distributed, maintaining registers, data compilation and entry<br>• Analyzing reported records and completing reports<br>• Technical working group or other meetings to discuss and report on data
Supplies and consumables	• Any infection, prevention and control materials used (masks, hand sanitizers, gloves, alcohol, biohazard bags) for on-site testing or training<br>• Any office supplies used for recording results etc. (pens, paper)

Scenario analysis

We calculated the total cost and the cost per self-test kit distributed for three main scenarios, shown in Table 3, that differed depending on the costing period used (actual pilot period compared to a theoretical one year implementation period), the number of tests distributed (actual, planned, scaled assuming an epidemic peak), the price of the self-test kit (pilot price, and a reduction of 50%); and the exclusion of staffing costs assuming that self-test kit distribution would be integrated with other routine services and the staff costs absorbed by the respective provider (workplace, healthcare facility).

Table 3. Three different implementation scenarios. Three different implementation scenarios with different sub-scenarios and other variables impacting the cost analyses

Scenario	Scenario description	Period	Number of tests	Cost
1	Pilot costing	Pilot period (preparation time + implementation)	Actual distribution as of 30th November 2022 extrapolated for the remaining period of the pilot	
2	Routine implementation on annual basis	Annual (12 months)	Planned distribution extrapolated to 12 months	50% reduction in the price of the test kit
3	Scaled-up peak implementation*	Annual (12 months)	Doubling of planned annual test distribution to accommodate an epidemic	50% reduction in the price of the test kit

For peer review only

For peer review only - http://bmjopen.bmj.com/site/about/guidelines.xhtml

			peak period (increased testing for the same number of beneficiaries)	Assume no staff costs**

*Assumed that staff did not have to increase to facilitate a doubling of tests distributed

**Assumed that staff costs would be absorbed by the respective provider (workplaces, or health facilities) and self-test kit delivery integrated into other activities

Ethics

The pilot projects in Pelotas and Afogados da Ingazeira, Brazil were conducted alongside routine operations at the targeted workplaces and thus did not require ethical approval. However, in accordance with country regulations for private data protection, written informed consent of participants was obtained prior to their enrollment. The socio-economic evaluation was designed, conducted, and reported in accordance with consolidated criteria for reporting health economic evaluations [19]. The Georgia study was approved by the National Center for Disease Control (NCDC) and Public Health Institutional Review Board (IRB) (Ref.: # 2022-049, May 24, 2022). The Malaysia study was approved by the Medical Research and Ethics Committee (MREC), Ministry of Health (MOH) Malaysia (Ref.: NMRR ID-22-02021-UCR, Oct 26, 2022). The pilot in Ethiopia was approved by the Ethiopian Public Health Institute Institutional Review Board (EPHI-IRB) (Ref.: EPHI-IRB-421 2022, Aug 19, 2022). The study in the Philippines was approved by Saint Cabrini Medical Center – Asian Eye Institute Ethics Review Committee (SCMC–AIE ERC) (Ref.: QR-ERC-002/05/00/12032020, Oct 19, 2022).

Patient and Public Involvement

Participants were involved in the design, conduct, and reporting of the research through inclusion in trainings, enrollment sessions, and reporting of data through various platforms as described in Table 1.

Results

Across all five countries, there were a total of 173,000 test-kits distributed during the pilot period (Table 4). Georgia had the largest pilot program (90,000 tests distributed); and Ethiopia the smallest (158 at the two healthcare facilities costed (this was scaled back from a planned distribution of 1250 kits). The cost per test distributed ranged from $2.44 (Malaysia) to $12.78 (Ethiopia). Despite having the lowest price per self-test kit purchased ($1.00), Ethiopia had the highest cost per test distributed ($12.78). This was due in part to low test volumes and higher resource intensity (i.e., staff time) associated with on-site testing at a

For peer review only - http://bmjopen.bmj.com/site/about/guidelines.xhtml

healthcare facility. Malaysia, in comparison, had the lowest cost per test distributed of $2.44, followed by public workplaces in Afogados da Ingazeira, Brazil ($2.82).

The cost per self-test kit distributed across all countries decreases with a routine implementation period of a year (Scenario 2 and 3) and with an increase in the scale of the program (Scenario 3) (Table 4). The cost per self-test kit distributed is lowest in scenario 3 with higher test demand (during peaks of incidence) assumed, and a test kit price reduction of 50%: ranging from $1.04 in Malaysia to $3.07 in Brazil (Pelotas). The total cost is however high for both Scenario 2 and 3 due to the high procurement costs associated with procuring more tests as well as the longer period of implementation, up to $1.7M for the largest program in Georgia. When assuming that self-test kit distribution would become a routinely integrated service, and assuming zero staffing costs, the cost per test for Scenario 3 ranged from $1.62 in the Philippines to $4.92 in Brazil (Pelotas).

Figure 1 summarizes the proportion of total costs that each cost category contributes to across scenarios and countries. Across all countries and scenarios, test procurement (the price of the self-test kits themselves including the costs shipping etc.) contributes the largest proportion and becomes even more important at greater scale (Scenario 3). Test procurement costs range from 58%–87% for countries with off-site testing (Georgia, Malaysia, Brazil), and 15% to 50% for countries with on-site testing (Ethiopia and the Philippines). Staff costs was the next key cost driver, particularly for distribution modalities that had on-site testing (29–35%) versus distribution modalities with off-site testing (7%–27%). Service delivery staffing costs (staff directly involved in the distribution of the self-test kits) ranged from as little as 3% in Malaysia (Scenario 3) to 26% in Ethiopia (Scenario 3). In the Philippines, Malaysia and both pilots in Brazil, staff management costs were the second largest cost driver ranging from 6% to 25% of total costs but decreasing with the scale of the program. This is particularly notable in the case of Ethiopia, which saw a sharp drop-off in the contribution of staff management costs when moving from a pilot program to more routine implementation as fixed management costs are spread across a higher number of tests.

For peer review only - http://bmjopen.bmj.com/site/about/guidelines.xhtml

Table 4. Program characteristics and all-inclusive cost per test for each country during the pilot (Scenario 1) and annual routine periods of implementation (Scenario 2 and 3

		Scenario 1 – pilot implementation				Scenario 2 – routine implementation			Scenario 3 – scaled peak implementation			
**Country**	**Testing location**	**Provider assisted or unassisted**	**Total tests distributed**	**Price of self-test kit**	**All-inclusive cost per test**	**Total tests**	**Current test-kit price:** Cost per test	**Reduced test-kit price:** Cost per test	**Total tests**	**Current test-kit price:** Cost per test	**Reduced test-kit price:** Cost per test	**Integrated staff:** Cost per test
**Brazil (Pelotas)**	Primarily off-site	Unassisted	10,000	$4.50	**$7.38**	20,000	**$6.54**	**$4.29**	40,000	**$5.62**	$3.37	**$4.92**
**Brazil (Afogados)**	Primarily off-site	Unassisted	26,000	$2.00	**$2.82**	52,000	**$2.59**	**$1.59**	104,000	**$2.30**	$1.30	**$2.06**
**Georgia**	Off-site	Unassisted	90,000	$2.00	**$4.44**	300,000	**$3.34**	**$2.34**	600,000	**$2.95**	$1.95	**$2.69**
**Malaysia**	Off-site	Unassisted	45,000	$1.55	**$2.44**	135,000	**$2.06**	**$1.28**	270,000	**$1.82**	$1.04	**$1.64**
**Ethiopia**	Off-site	Provider assisted	158	$1.00	**$12.78**	5,040	**$4.22**	**$3.72**	10,080	**$3.80**	$3.30	**$2.56**
**Philippines**	Off-site	Unassisted	2,000	$1.32	**$4.99**	6,000	**$4.51**	**$3.85**	12,000	**$2.92**	$2.26	**$1.62**

Figure 1: Cost drivers and the cost per test distributed by country and scenario at current self-test kit procurement prices

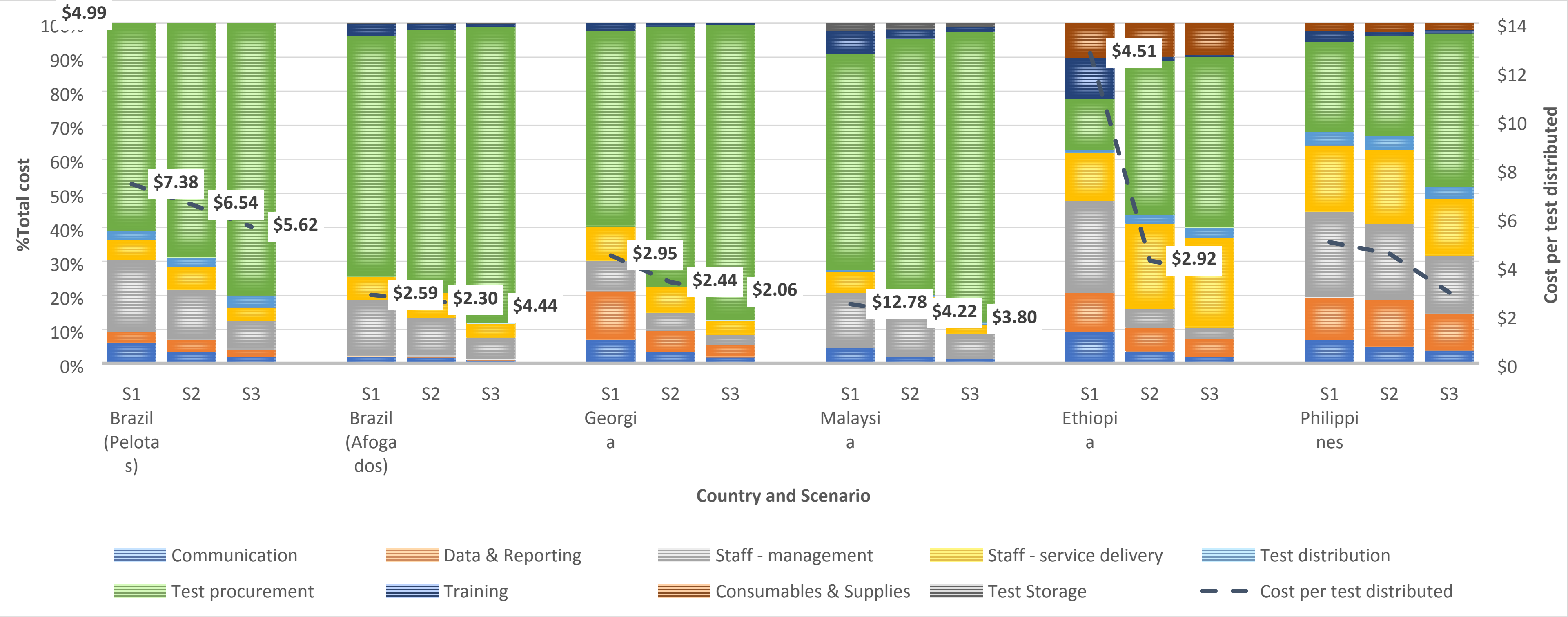

14

Discussion

We aimed to determine the cost per COVID-19 self-test allocated by distribution modality as well as the cost per COVID-19 self-test performed. Across all five countries analyzed in this study, there were a total of 173,000 kits distributed with the cost per test distributed ranging from \$2.44–\$12.78. The cost per self-test kit distributed is lowest in the scenario that assumed implementation over a longer period (one year), with higher test demand (peak), and a test kit price reduction of 50% (\$1.04–\$3.07).

COVID-19 self-tests are generally cost-saving compared to PCR-assays and professional use Ag-RDTs. The average cost of an automated PCR-assay is at least \$10.00, excluding the cost of sample collection or result delivery [2]. In comparison, the cost of a COVID-19 self-test in the pilot studies ranged from \$1.00 to \$4.50 making them a more affordable option for expanding access to testing and thereby increasing detection of COVID-19 cases and enacting recommended preventative measures [2]. Despite similarities across countries in terms of income categorization (three countries are categorized as MICs) as well as distribution modality (five out of the six were workplace distribution models), the cost of *distributing* COVID-19 self-tests varied substantially. This variability in cost can be attributed to the type of COVID-19 self-test that were used (as shown above), the amount of self-tests that were procured, and the modality of distribution or pilot implementation methodology. We found that across countries and scenarios, test procurement (the price of the self-test kit and shipping) constitutes the highest proportion of the total costs of distribution: 58% to 87% for countries with off-site testing and 15% to 50% for countries with on-site testing. Whilst this might suggest that country governments have little flexibility in influencing the overall cost of distributing COVID-19 self-tests outside of procurement negotiations, there are still substantial costs related to the delivery of COVID-19 tests and as such potential for cost reduction, particularly through the design of the distribution modality itself (for example, on-site versus off-site testing). Additionally, the substantial cost of test procurement and shipping may indicate that purchasing COVID-19 self-tests from regional manufacturers or better supply management and coordination may help reduce costs.

We can see that tests that were performed on-site resulted in a greater unit cost per self-test compared to tests that were performed off-site. For example, the on-site pilot implemented in healthcare facilities in Ethiopia (\$12.78), required a significantly greater amount of staff-time and consumables when compared to the off-site pilot implemented in Malaysia (\$2.44). We would expect to see further deviation in the cost of distribution of self-tests in both HICs and LMICs as donor funding and access to Ag-RDTs differs in settings with high and low resources respectively. Additionally, we can see that the cost to distribute COVID-19 self-tests can be expected to differ during routine program implementation rather than pilot

For peer review only - http://bmjopen.bmj.com/site/about/guidelines.xhtml

implementation. We attempted to estimate how start-up costs and pilot staff planning and management costs dedicated to the self-test program would be reduced during routine implementation (Scenarios 2 and 3). This fluctuation in cost can be described as the "U-shape phenomenon"; the cost per test distributed is initially high but decreases as the program scales-up distribution since fixed start-up (data systems, planning and communication design) and once-off costs (training) are spread over a greater number of tests. Further, we even showed how this cost is further reduced when the distribution of self-test kits is completely integrated into routine operations and the staffing costs are incrementally small and absorbed by the provider. However, at a certain level of scale, the cost per test will increase again as diseconomies of scale initiate as well as when additional staff and training is required to facilitate the distribution of more and more tests.

Prior to this study, little was known about the cost to implement COVID-19 self-testing programs, however, self-test distribution has been offered via different modalities for various infectious diseases, including HIV, in which we can draw some comparisons. *Sande et al.* found that the key cost drivers of offering HIV self-test kits at public health facilities across four sub-Saharan African countries were personnel and the cost per test kit [6]. This was largely true across distribution modalities and countries for COVID-19 self-testing, as test procurement comprised the largest contribution to total costs, followed by staff management and service delivery categories, even though the cost per test procured differed by country. In the analysis by *Sande et al.*, they observed economies of scale across sites, as the cost per HIV self-test kit distributed was lower among sites that distributed more test kits, with the cost ranging from \$4.27–13.42 [6]. We observed a similar trend in our analysis when scaling up the number of COVID-19 self-test kits distributed, assuming staff costs remain consistent, and start-up related costs begin to taper off, with increased distribution. In another analysis by *Matsimela et al.* that looked at HIV self-test kit distribution across 11 modalities in South Africa, including workplaces, they observed economies of scale related to test kit distribution volume. Test kit cost and personnel were indeed the greatest cost drivers in the workplace modalities in that analysis, with the cost to distribute an HIV self-test in the workplace ranging from \$5.30–6.29 [10]. While we cannot directly compare the cost to distribute an HIV self-test kit to a COVID-19 self-test kit due to the inherently different nature of the two viruses' epidemiology (COVID-19 can require more frequent repeat testing), as well as the nature and stigmatization of diagnosing HIV, we do see a similar range in the cost per COVID-19 self-test kit distributed from \$2.44–12.78. Our analysis provides an overview of the potential overall costs to offer COVID-19 self-testing programs.

To our knowledge, this study is the first to estimate the costs of COVID-19 self-test distribution across different countries and settings. We determined the costs based on the best available evidence from pilot

For peer review only - http://bmjopen.bmj.com/site/about/guidelines.xhtml

studies conducted in five heterogeneous countries across predominately workplace distribution modalities. There are however several limitations. Firstly, whilst we tried to exclude study-specific costs, there might be protocol-induced higher resource-use costs – which may not be observed at scale-up (including higher uptake of testing and reporting due to pilot-specific follow-up). This might result in higher total costs, but also more tests performed potentially resulting in a net effect on the cost per test. Second, we did not conduct a time in motion analysis to assess the time spent by each staff cadre on pilot-related activities. As such, it is probable that our estimates on the average time spent by staff on pilot activities per month are overestimated. It is likely that the time spent on test kit distribution will decrease after the starting months, and especially with routine implementation. We tried to adjust for this by excluding all staff costs under Scenario 3 to provide a lower bound of what this cost might be. Third, in Scenario 3 we have assumed that there would be no need for additional staff or staff time if the number of tests distributed were to double. However, since it is likely that we overestimated the average time spent by staff on pilot activities per month, we felt that this assumption was reasonable. Fourth, in some countries (Brazil and Malaysia), the pilot was ongoing, and we had to extrapolate figures (actual COVID-19 test distribution), as well as use budgeted expenditure instead of actual expenditure. Fifth, across countries, actual distribution was lower than planned distribution due to low COVID-19 prevalence. The stage of the epidemic would affect the uptake of self-tests and subsequently the cost per test. To account for this, Scenario 3 assessed a doubling of testing demand as a result of an assumed peak in the epidemic to approximate high demand or uptake phases. Lastly, outside of the workplace distribution model, the only other modality piloted was the on-site testing at healthcare facilities in Ethiopia. As such, we were unable to understand the relative difference in implementation costs across modalities limiting the generalizability of these costs to other distribution modalities.

Conclusion

Our results indicate that it is likely to cost between \$2.44 and \$12.78 per test to distribute COVID-19 self-tests across common settings in five heterogeneous countries with differing economical characteristics, health systems governance, and epidemiological and demographic profiles. Additional costing and cost-effectiveness analyses based on these results will allow policy makers to make informed decisions on optimal approaches to scaling up COVID-19 self-test distribution programs across settings and countries as epidemiology and testing needs continue to evolve.

For peer review only - http://bmjopen.bmj.com/site/about/guidelines.xhtml

References

1. WHO. Use of SARS-CoV-2 antigen-detection rapid diagnostic tests for COVID-19 self-testing. Interim Guide. 2022; 1–16.

2. The Global Fund. Pooled Procurement Mechanism Reference Pricing: COVID-19 diagnostics 2022. Available: https://pfscm.org/wpcontent/uploads/2021/12/PFSCM_COVID_PCR_Guidance_2021.pdf

3. Han AX, Girdwood S, Khan S, Sacks JA, Toporowski A, Huq N, et al. Strategies for using antigen rapid diagnostic tests to reduce transmission of SARS-CoV-2 in low- and middle-income countries: a mathematical modelling study. medRxiv. [cited 25 Oct 2022] Available: https://www.medrxiv.org/content/10.1101/2022.06.16.22276516v1

4. Hoehl S, Schenk B, Rudych O, Göttig S, Kohmer N, Karaca O, et al. High-Frequency Self-Testing by Schoolteachers for Sars-Cov-2 Using a Rapid Antigen Test: Results of the Safe School Hesse study. Dtsch Arztebl Int. 2021. doi:10.3238/arztebl.m2021.0187

5. Papenburg J, Campbell JR, Caya C, Dion C, Corsini R, Cheng MP, et al. Adequacy of Serial Self-performed SARS-CoV-2 Rapid Antigen Detection Testing for Longitudinal Mass Screening in the Workplace. JAMA Netw Open. 2022;5: e2210559. doi:10.1001/jamanetworkopen.2022.10559

6. Sande LA, Matsimela K, Mwenge L, Mangenah C, Choko AT, D'Elbée M, et al. Costs of integrating HIV self-testing in public health facilities in Malawi, South Africa, Zambia, and Zimbabwe. BMJ Glob Heal. 2021;6: 1–9. doi:10.1136/bmjgh-2021-005191

7. Martínez-Pérez GZ, Shilton S, Saruê M, Cesario H, Banerji A, Batheja D, et al. Self-testing for SARS-CoV-2 in São Paulo, Brazil: results of a population-based values and attitudes survey. BMC Infect Dis. 2022;22: 720. doi:10.1186/s12879-022-07706-7

8. Nwaozuru U, Obiezu-Umeh C, Diallo H, Graham D, Whembolua GL., Bourgeau MJ, Ritchwood TD, et al. Perceptions of COVID-19 self-testing and recommendations for implementation and scale-up among Black/African Americans: implications for the COVID-19 STEP project. BMC Public Heal. 2022; 22: 1. doi:10.1186/s12889-022-13619-6

9. Jamieson L, Johnson LF, Matsimela K, Sande LA, D'Elbée M, Majam M, et al. The cost effectiveness

For peer review only - http://bmjopen.bmj.com/site/about/guidelines.xhtml

and optimal configuration of HIV self-test distribution in South Africa: A model analysis. BMJ Glob Heal. 2021;6: 1–10. doi:10.1136/bmjgh-2021-005598

10. Matsimela K, Sande LA, Mostert C, Majam M, Phiri J, Zishiri V, et al. The cost and intermediary cost-effectiveness of oral HIV self-test kit distribution across 11 distribution models in South Africa. BMJ Glob Heal. 2021;6: 1–10. doi:10.1136/bmjgh-2021-005019

11. Walker JG, Ivanova E, Jamil MS, Prof A, Ong JJ, Easterbrook PP, et al. Cost and cost-effectiveness of Hepatitis C virus self-testing in four settings: an economic evaluation. 2022.

12. National Bank of Ethiopia. Inter-Bank Daily Foreign Exchange Rate - National Bank. [cited 25 Nov 2022]. Available: https://nbe.gov.et/inter-bank-daily-foreign-exchange-rate-in-usd/

13. National Bank of Georgia. Statistics Data: Exchange Rates. [cited 25 Nov 2022]. Available: https://nbg.gov.ge/en/statistics/statistics-data?title=&code=

14. The Central Bank of Brazil. Exchange rates: Quotations and Bulletins. [cited 25 Nov 2022]. Available: https://www.bcb.gov.br/en/financialstability/quotations%0A

15. The Central Bank of Malaysia. Rates and Statistics: Exchange Rates. [cited 25 Nov 2022]. Available: https://www.bnm.gov.my/exchange-rates

16. The Central Bank of the Republic of Philippines. Statistics: Exchange Rate. [cited 25 Nov 2022]. Available: https://www.bsp.gov.ph/SitePages/Statistics/ExchangeRate.aspx

17. Fuel economy in Brazil IEA. Fuel Economy in Brazil. [cited Jan 2023]. Available: https://www.iea.org/articles/fuel-economy-in-brazil

18. Boonstoppel L, Banks C, Moi F, Vaughan K, Ozaltin A, Brenzel L. How to Conduct an Immunization Campaign Costing Study: Methodological Guidance. 2021. Washington, DC: ThinkWell.

19. Husereau D, Drummond M, Petrou S, Carswell C, Moher D, Greenberg D, et al. Consolidated Health Economic Evaluation Reporting Standards (CHEERS) statement. 2013. BMJ; 346 :f1049 doi:10.1136/bmj.f1049

For peer review only

For peer review only - http://bmjopen.bmj.com/site/about/guidelines.xhtml

396 **Acknowledgements**

397 We would like to thank all the in-country study teams and implementing partners for their assistance with
398 acquiring data: Brazil (Antakly: Gisela Antakly and Joselito Pedrosa and Germsure: Ana Flavia Pires and
399 Cassia Gonçalves, Pelotas: Lifemed, Afogados da Ingazeira: Municipal Health Secretary); Malaysia
400 (FIND: Xiao Hui Sem and Farhana Zulkifli; MOH of Malaysia: Dr Sunita Binti Abdul Rahman and Dr
401 Chan Huan-Keat); Ethiopia (KNCV: Lensa Chala and Israel Mitiku); Philippines (KNCV: Robert Leong);
402 Georgia (FIND: Maia Japaridze and Ia Jikia; National Center for Disease Control); and all sites participating
403 in the pilots' implementation.

404

405 **Author Contributions**

406 Conceptualization: BEN Data curation: PDRP, EMC, GZMP, LC, RL Formal analysis: MH, NL, JMC, TO,
407 SJG Investigation: MH, NL, JMC, TO, SJG Methodology: MH, NL, JMC, TO, SJG Supervision: SJG,
408 BEN Writing - original draft: MH, NL, JMC, TO, SJG, BEN Writing – review and editing: MH, NL, JMC,
409 TO, PDRP, EMC, GZMP, SS, MRAH, VG, CWM, MITB, LC, RL, RP, SK, IS, CJ, SJG, BEN

410

411 **Competing Interests**

412 The authors declare no conflict of interest. The funders had no role in the design of the study; in the
413 collection, analyses, or interpretation of data; in the writing of the manuscript; or in the decision to publish
414 the results.

415

416 **Funding**

417 This costing study was funded by KfW Group, the German state-owned investment and development bank;
418 grant number KfW-TBBU02. The funders played no role in the design of the study; the collection,
419 management, analysis, or interpretation of data; writing of the report; or the decision to submit the report
420 for publication.

421

422 **Data Availability Statement**

423 Anonymized cost data is available.

424

425 **Appendix**

426 Detailed costing tables for each distribution modality, indicating costing assumptions and describing costs
427 included.

For peer review only - http://bmjopen.bmj.com/site/about/guidelines.xhtml

Appendix / Supplementary Materials

Table A1. Cost categories with descriptions for Pelotas, Brazil. For every cost category, all costs included, and any assumptions made are listed. All costs are reported in 2022 USD.

**Cost category**	**Description & explanation**
Test procurement	• GeneFinder COVID-19 Ag Self-Test from Osang Healthcare (South Korea)<br>• Self-test unit price (including shipping/importation, taxes, duties, custom clearance): $4.50<br>• 10,000 self-tests procured
Test distribution	• Single shipment from Sao Paulo, Brazil to the workplace storage facility in Pelotas, Brazil at a cost of $0.19 per test.
Test storage	• Tests were stored on-site at the workplace and incurred no additional costs for storage space.<br>• Costs included were employee time required to rearrange storage facility contents to accommodate self-test shipment (2 x staff, 2 hours each x salary per hour).
Training	• Training of pilot beneficiaries on program and proper self-test use.<br>• Training costs were directly related to staff time/salary and are included in staff-management and staff-service delivery cost categories.
Communication	• All communication materials were digital and included posters, banners, and videos to promote COVID-19 self-testing to beneficiaries.<br>• Costs included under communication were for the design of materials (hourly cost of designer and consultant: 80 hours x $40/hour and 60 hours x $20/hour).
Staff: Management	• Project implementation support is a combination of internal employees and external partners.<br>• Four internal workplace employees with partial dedication (between 4-8 hours per month) and one external consultant (allocated 127 hours for the period of the pilot).<br>• Costs include staff-time and salary dedicated to management, oversight and support of the testing program and the program manager.
Staff: Service delivery	• 1 person was hired to be a dedicated full-time program manager of the testing program for the duration of the pilot.<br>• Duties include enrolment, training, test distribution, and data collection/management.
Data and reporting	• Self-test results are reported to the program manager and uploaded to the digital platform.<br>• The cost of the digital platform comprises development costs incurred by IT personnel (80 hours multiplied by staff hourly rate).<br>• Data and reporting costs also include the cost to host meetings/working groups four times over the course of the pilot for 7 people on average at a cost of $57 per person per meeting and $150 per meeting (venue, catering).<br>• Staff time costs that relate activities conducted for data and reporting are included under the staff cost categories.
Consumables and supplies	• Not applicable for this pilot, as testing is performed off-site.
Other	• The pilot was conducted at one site. The pilot took place from September 2022 to February 2023. Costing and data collection was completed in December 2022; therefore, some costs and test results were extrapolated for the remaining 2 months of the pilot.

For peer review only - http://bmjopen.bmj.com/site/about/guidelines.xhtml

Table A2. Cost categories with descriptions for Afogados da Ingazeira, Brazil. For every cost category, all costs included, and any assumptions made are listed. All costs are reported in 2022 USD.

Cost category	Description & explanation
Test procurement	• COVID-19 self-test kit: Alerta COVID-19 Ag Self-Test manufactured by WAMA Diagnóstica based in Sao Carlos, Brazil<br>• Self-test unit price (including shipping, taxes, duties, custom clearance): $2.00<br>• 26,000 self-tests procured
Test distribution	• Tests were moved from a central storage facility to the Education and Health Secretariat offices using a municipal transport system at no extra cost.<br>• Tests were distributed from Education and Health Secretariat offices to the respective managers of each of the 34 sites included in the pilot study. During the time of the pilot, fuel costs were observed to be $1/liter. Considering the fuel economy for a light duty vehicle in Brazil and maintenance expenses, transportation of 26,000 self-tests to all 34 sites was calculated to be $850.
Test storage	• Tests were stored at an existing storage facility that was centrally located to the Education and Health Secretariats where they were delivered by the manufacturer. No additional cost was incurred from use of the storage space.<br>• 4 palletes to place the boxes holding self-tests were purchased at $37.10 each.
Training	• 2 training workshops were hosted and catered for, with a session providing instructions on how to use the self-test. $400 was budgeted for each training workshop.<br>• A training guide was provided to pilot staff to sensitise staff regarding different self-test procedures as courtesy of the test manufacturer (no cost associated).<br>• 12 hours of initial training hosted by an external consulting agency for a fixed once-off cost of $1656.<br>• Subsequent training sessions on self-test distribution and use were hosted online at no extra cost.
Communication	• Three review meetings were hosted and catered to discuss and communicate the launch of the pilot, progress, and results of the self-test pilot. $400 was budgeted for each review meeting.<br>• Advertisement posters, pamphlets, and stickers were designed and distributed to the beneficiaries at initiation. The communication materials explain when to use tests and what to do depending on the result. The materials cost $220.
Staff: Management	• Project implementation support is provided by two external consulting agencies at a cost of $552 USD per month, as well as a fixed once-off cost of $7,112.
Staff: Service delivery	• 34 individuals comprising four different working groups (operational team, monitoring team, quality control team and management team) contributed approximately 5% of their working time to the project at a cost of $24.29 per individual per month.
Data and reporting	• Self-tests were recorded by beneficiaries in a Google Form that was sent out weekly. No cost was associated with the distribution and design of the Google Form.<br>• Data was managed by two site managers at a rate of $30 per individual per month.
Consumables and supplies	• Not applicable for this pilot, as testing is performed off-site.
Other	• The pilot was conducted at a total of 34 sites. The pilot took place during October 2022 to March 2023. Costing and data collection was completed in December 2022; therefore, some costs and test results were extrapolated for the remaining 3 months of the pilot.

For peer review only - http://bmjopen.bmj.com/site/about/guidelines.xhtml

Table A3. Cost categories with descriptions for Georgia. For every cost category, all costs included, and any assumptions made are listed. All costs are reported in 2022 USD.

Cost category	Description & explanation
Test procurement	● COVID-19 self-test kit: On-site COVID-19 Ag Self-Test® (CTK Biotech, California, USA)<br>● Self-test unit price (including shipping, taxes, duties): $2.00<br>● 90,0000 self-tests procured
Test distribution	● Fuel for pilot staff to transport tests from the National Centres for Disease Control (NCDC) to Primary Healthcare Center in Mestia, and from there to schools in the Svaneti region.<br>● Only costs for transportation of tests to schools are accounted for. The NCDC already makes regular trips to the health care facilities and nursing homes for delivery of other supplies through an established delivery system. The additional cost, as well as the opportunity cost of distributing self-tests was considered negligible.
Test storage	● No additional storage space had to be rented to store tests. Every site owned a storage of their own where tests were stored and there was no opportunity cost to the storing of tests.<br>● No additional supplies were purchased to store tests.<br>● Storage of the tests at NCDC was considered negligible.
Training	● A workshop for each of the modalities was provided to train staff (facilitators, principal investigators, project managers) to perform actions needed in the pilot. Costs for these workshops consisted of fuel for transportation, flights (for the school modality), printing supporting materials, supplies (hand sanitizers and masks), venue and hotel costs. - a total of 5 trainings<br>● Allocation of shared training costs for schools, nursing homes and hospital/clinic modalities was based on the number of facilitators allocated to the different modalities.
Communication	● NCDC Communication staff (marketer, social media manager, designer, health promotion, social media coordinator).<br>● Making of videos, social media posts etc. on self-testing and general testing.<br>● Pens, bags, brochures, posters, banners.
Staff: Management	● Salary cost of time spent by:<br>○ Principal investigators (PI and co-PI) at NCDC<br>○ Project managers (PM) at NCDC as well as sites (total of 15)<br>○ Project implementation and support including consultant time<br>○ Finance and admin support from NCDC<br>● Used average Georgian salaries for the current staff position (instead of a stipend paid to staff by FIND to conduct the pilot) to ensure better alignment with the actual costs of scaling up test distribution.<br>● Estimated the average proportion of their time each month that is dedicated to COVID-19 self-test implementation and operations.<br>● Pilot-related staff, such as PMs included as time largely spent on implementation (with a small component spent on research-specific activities).<br>● Included 2 months preparation time for the pilot.
Staff: service delivery	● This includes the salary of the facilitators at each of the sites (NCDC facilitators (9), hospitals (24), clinics (7), nursing homes (4), and schools (24), and then the average proportion of their time each month that is dedicated to COVID-19 self-test operations.<br>● Facilitator activities included:<br>○ Facilitator time spent on collecting COVID-19 self-tests to bring to appropriate testing facility (i.e., workplace, schools)<br>○ Facilitator time spent on storing and organizing COVID-19 self-tests<br>○ Facilitator time spent on distributing the COVID-19 self-test to participants<br>○ Facilitator time spent on training clients/participants on how to use the COVID-19 self-test, including information and sensitization activities

For peer review only - http://bmjopen.bmj.com/site/about/guidelines.xhtml

	• Used average Georgian salaries for the current staff position (instead of a stipend paid to staff by FIND to conduct the pilot) to ensure better alignment with the actual costs of scaling up test distribution.
Data and reporting	• Costs associated with the development and use of a database/app where results are documented and reported.<br>• Staff time of data entry operators at hospitals (6), clinics (2) and nursing homes (2). (Facilitators conduct data entry at schools). Data entry operators are responsible for record keeping after COVID-19 self-tests are distributed, maintaining registers, data compilation and entry. Beneficiaries test weekly and, if positive, call in immediately for the data operator to record it. If negative, the beneficiary will submit the result at work.<br>• Technical Working Group meetings to report and share results (venue, food, IT, and transportation) – a total of 3.<br>• Data support from the NCDC in terms of staff time (5).

For peer review only

For peer review only - http://bmjopen.bmj.com/site/about/guidelines.xhtml

Table A4. Cost categories with descriptions for Malaysia. For every cost category, all costs included, and any assumptions made are listed. All costs are reported in 2022 USD.

**Cost category**	**Description & explanation**
Test procurement	• COVID-19 self-test kit: FlowFlex Ag-RDTs manufactured by ACON Biotech (Hangzhou) Co., Ltd.<br>• Self-test unit price (including shipping, taxes, duties, custom clearance): $1.55<br>• 45,000 self-tests procured
Test distribution	• Tests were shipped from the central storage facility to each site through a third-party courier at a rate of $0.0121 per test. This cost included door-to-door delivery, driver salary, fuel, vehicle, etc.<br>• For the 45,000 tests procured for the pilot, test distribution costs totalled $545.
Test storage	• Tests were stored in a central storage facility in Kuala Lumpur at a cost of $420 per month.<br>• A temperature data logger was purchased to ensure temperature regulation of the storage facility as a once off cost of $20.
Training	• 2 in-person training sessions were hosted for a research assistant dedicated to the self-testing pilot. Travel associated with attending each session totalled $286.<br>• 1 training session was hosted at each site (4 total) to train site managers on the self-testing pilot. $915 was incurred for each training session per site.<br>• A sensitization session was hosted at each site (4 total) to train beneficiaries on how to use the FlowFlex Ag-RDT. $458 was incurred for each training session per site.
Communication	• Supplementary information and reference guides for the self-tests were provided to each beneficiary. $1030 was budgeted for these communication materials.<br>• 3 working group meetings were hosted to monitor and communicate upon the reception and progress of the COVID-19 self-testing pilot. $1145 was budgeted for each working group meeting.
Staff: Management	• Project implementation support is provided by an external organisation for $1438 per month.
Staff: Service delivery	• A research assistant was dedicated to delivering the self-tests to their respective sites and hosting the sensitization sessions for the beneficiaries. Salary and travel expenses for this position resulted in a total cost of $1145 per month.
Data and reporting	• Self-tests were recorded by beneficiaries in a Google Form that was sent out weekly and managed by the project's dedicated research assistant. No additional cost was associated with the distribution and management of the Google Form.<br>• A sim card was purchased to follow-up with beneficiaries via phone who did not report their test results on the Google form for $11 per month.
Consumables and supplies	• Not applicable for this pilot, as testing is performed off-site.
Other	• The pilot was conducted at a total of 2 sites. The pilot took place during November 2022 to April 2023. Costing and data collection was completed in December 2022; therefore, some costs and test results were extrapolated for the remaining 2 months of the pilot.

For peer review only - http://bmjopen.bmj.com/site/about/guidelines.xhtml

Table A5. Cost categories with descriptions for Ethiopia. For every cost category, all costs included, and any assumptions made are listed. All costs are reported in 2022 USD.

Cost category	Description & explanation
Test procurement	• COVID-19 self-test kit: Flowflex Acon Biotech self-test kit, ACON Biotech (Hangzhou) Co.,Ltd.<br>• Self-test unit price: $1.00<br>• 1,250 self-tests procured
Test distribution	• Transportation cost from the airport to health care facilities. Once-off cost ($140) for all imported tests used to estimate a cost per self-test kit transported.
Test storage	• No additional storage space had to be rented to store tests. Every site owned a storage of their own where tests were stored and there was no opportunity cost to the storing of tests.<br>• No additional supplies were purchased to store tests.
Training	• A video was produced and procured for self-test kit demonstration purposes. Field coordinators initially watched the video themselves (as part of training). The video was also shown to patients to demonstrate proper self-test kit usage.<br>• Once-off cost of $500 for video production.
Communication	• Mobile card usage for four field coordinators (2 per site) to provide updates on the study.<br>• Monthly cost per mobile card: $14.81
Staff: Management	• Salary cost of time spent by:<br>○ Cleaner, administrative assistant, monitoring & evaluation officer, and program coordinator at sites (total of 8 sites)<br>○ Project implementation and support including consultant time from implementing partner<br>○ Finance and administration support from implementing partner<br>• Used average Ethiopian salaries for the current staff position (instead of a salary paid by the implementing partner to conduct the pilot) to ensure better alignment with the actual costs of scaling up test distribution.<br>• Estimated the average proportion of their time each month that is dedicated to COVID-19 self-test implementation and operations.<br>• Allocated the time of all staff to the 2 sites used in the costing analysis based on test-kit distribution For the routine implementation scenario analysis, the level of effort of the monitoring and evaluation officer was reduced, and the salary cost of the monitoring and evaluation officer and programme coordinator was aligned with that of the staff cadre that would likely fill these positions should the programme be scaled.
Staff: Service delivery	• This includes the salary of the OPD physicians (1 per site), nurses and field coordinators (2 per site) at each of the sites (2 sites).<br>• Staff: service delivery activities included:<br>○ OPD physicians: time spent on evaluation clients if they are eligible for self-testing and linking them to field coordinators (1 hour per day)<br>○ Nurses: time spent on observing clients execute self-testing, collect, and process the sample and finally read the result<br>○ Field coordinators: time spent on arrival and registration of client, showing client a demonstration video, pre-test counselling, preparing the testing area and conclude by providing post-test counselling/information<br>• The direct staff cost for nurses and field coordinators was estimated on a per test basis by observing time estimates spent on service delivery and salary cost per minute: staff nurse (25 minutes per client); field coordinator (42 minutes per client).

For peer review only - http://bmjopen.bmj.com/site/about/guidelines.xhtml

	• Used average Ethiopian salaries for the staff cadre required to perform the task (instead of a salary paid to staff by the implementing partner to conduct the pilot) to ensure better alignment with the actual costs of scaling up test distribution.
Data and reporting	• Field coordinators provide post-counselling/information to clients and document and report the self-test result.<br>• Unlimited internet SIM card procured for four field coordinators (acting as data collectors; 2 per site) for facilitation of data entry on REDCap: $16.67 per month.
Consumables and supplies	• Costed hand sanitizer, gloves, and alcohol for disinfecting the testing area on a per test basis.<br>• Costed masks and biohazard bags used by the facility and staff to oversee the self-testing.
Other	• The pilot was conducted at a total of 8 sites. However, given costing timelines, costs were only collected for 2 sites. These sites are however representative of the other sites.<br>• Costs for the video production and overhead staff management were allocated to the 2 sites based on test-kit distribution (46% of test kits were distributed at the 2 sites that were costed).

For peer review only - http://bmjopen.bmj.com/site/about/guidelines.xhtml

Table A6. Cost categories with descriptions for the Philippines. For every cost category, all costs included, and any assumptions made are listed. All costs are reported in 2022 USD.

Cost category	Description & explanation
Test procurement	• COVID-19 self-test kit: Flowflex Acon Biotech self-test kit, ACON Biotech (Hangzhou) Co.,Ltd.<br>• Self-test unit price: $1.32 (including shipment of test from the local distributor – taxes and duties)<br>• 2,000 self-tests procured
Test distribution	• Transport billed in bulk inclusive of porter's/driver's salary, vehicle rental, and applicable fuel, toll fees, and taxes. Fixed cost per day, per site, per month.<br>• Tests are distributed directly to the partner sites.
Test storage	• No additional storage space had to be rented to store tests at the central office. Store to capacity and re-order when need arises.<br>• Each site has storage of its own and no opportunity cost to the storing of tests.
Training	• A 1-day training workshop was conducted with all partner site staff (50 in total).<br>• Each participant received 2 demonstration tests.<br>• A cost per person trained per day was calculated to estimate the cost of training the staff at one site.
Communication	• All site staff receive a monthly communication allowance inclusive of phone, internet service provider subsidy and data transfer to servers.<br>• Management staff also receive a monthly communication allowance to manage all 7 sites. This cost is allocated to the costed site using 1/7 factor.<br>• Printing papers, stationery, cartridges for brochures, Information Education Communication (IEC) materials, and posters, banners, and brochures – once-off per site.
Staff: Management	• 1 x Project implementer Research officer (supporting all sites).<br>• 1x Project M&E Officer – solely contracted by project implementer to supervise across all sites.<br>• 1 x Site Supervisor (clinic staff) at nurse salary grade 19 (government salary grade)<br>• Estimated the average proportion of their time each month that is dedicated to COVID-19 self-test implementation and operations:<br>○ 5 hours per 8-hour day for the Project implementer Research officer<br>○ 5 hours per 8-hour day for the Project M&E Research officer<br>○ 1 hour per 8-hour day for the Site Supervisor (clinic staff)<br>• Research staff time spent on self-test distribution allocated per site (monthly salary/7).<br>• M&E Research staff time spent on self-test distribution allocated per site (monthly salary/7).<br>• Included 1-month of preparation time.
Staff: Service delivery	• 1x Test counsellor –at medical technologist II salary grade 15 (government salary grade).<br>• 1x Test administrator – at Nurse Salary I with salary grade 15.<br>• Estimated the average proportion of their time each month that is dedicated to COVID-19 self-test implementation and operations (3 hours per 8-hour day).
Data and Reporting	• 1x Site Data Entry Operator – Project implementer assigned staff on-site, Nursing Attendant II w/ Salary Grade 6 (government salary grade).<br>• Estimated the average proportion of their time each month that is dedicated to COVID-19 self-test implementation and operations:<br>○ 100% of their time for the Data Entry Operator

For peer review only - http://bmjopen.bmj.com/site/about/guidelines.xhtml

Consumables and Supplies	• Hygiene essentials: infection control kits available on-site as part of IPC procedures (alcohol, masks, gloves, hand soap, paracetamol, disinfectants, thermometer).<br>• Office supplies for staff: documentation and office essentials (printing papers, stationery, cartridges, ball pens).
Other	• The pilot was conducted at a total of 7 sites. Only 1 site was costed. This site was considered typical of the other sites; set-up is the same across sites, only the scale of testing differs.<br>• Costs for the staff that oversee operations across sites, their communication allowance and the supplies and consumables were allocated to one site based on the factor of 1/7.

For peer review only

CHEERS 2022 Checklist

Topic	No.	Item	Location where item is reported
**Title**			
	1	Identify the study as an economic evaluation and specify the interventions being compared.	Title
**Abstract**			
	2	Provide a structured summary that highlights context, key methods, results, and alternative analyses.	Abstract
**Introduction**			
**Background and objectives**	3	Give the context for the study, the study question, and its practical relevance for decision making in policy or practice.	Introduction, start at line 106
**Methods**			
**Health economic analysis plan**	4	Indicate whether a health economic analysis plan was developed and where available.	Methods, start at line 133 and 158
**Study population**	5	Describe characteristics of the study population (such as age range, demographics, socioeconomic, or clinical characteristics).	Methods, start at line 115
**Setting and location**	6	Provide relevant contextual information that may influence findings.	Methods, start at line 126
**Comparators**	7	Describe the interventions or strategies being compared and why chosen.	Methods, start at line 158
**Perspective**	8	State the perspective(s) adopted by the study and why chosen.	Methods, line 133
**Time horizon**	9	State the time horizon for the study and why appropriate.	Methods, line 134-135
**Discount rate**	10	Report the discount rate(s) and reason chosen.	Methods, line 136-137
**Selection of outcomes**	11	Describe what outcomes were used as the measure(s) of benefit(s) and harm(s).	Not applicable
**Measurement of outcomes**	12	Describe how outcomes used to capture benefit(s) and harm(s) were measured.	Not applicable

For peer review only - http://bmjopen.bmj.com/site/about/guidelines.xhtml

Topic	No.	Item	Location where item is reported
**Valuation of outcomes**	13	Describe the population and methods used to measure and value outcomes.	Not applicable
**Measurement and valuation of resources and costs**	14	Describe how costs were valued.	Methods, line 133
**Currency, price date, and conversion**	15	Report the dates of the estimated resource quantities and unit costs, plus the currency and year of conversion.	Methods, line 136-137
**Rationale and description of model**	16	If modelling is used, describe in detail and why used. Report if the model is publicly available and where it can be accessed.	Not applicable
**Analytics and assumptions**	17	Describe any methods for analysing or statistically transforming data, any extrapolation methods, and approaches for validating any model used.	Methods, line 158
**Characterising heterogeneity**	18	Describe any methods used for estimating how the results of the study vary for subgroups.	Methods, starting at line 158
**Characterising distributional effects**	19	Describe how impacts are distributed across different individuals or adjustments made to reflect priority populations.	Methods, starting at line 149
**Characterising uncertainty**	20	Describe methods to characterise any sources of uncertainty in the analysis.	Methods, line 158
**Approach to engagement with patients and others affected by the study**	21	Describe any approaches to engage patients or service recipients, the general public, communities, or stakeholders (such as clinicians or payers) in the design of the study.	See appendices
**Results**			
**Study parameters**	22	Report all analytic inputs (such as values, ranges, references) including uncertainty or distributional assumptions.	Not applicable
**Summary of main results**	23	Report the mean values for the main categories of costs and outcomes of interest and summarise them in the most appropriate overall measure.	Results, Figure 1
**Effect of uncertainty**	24	Describe how uncertainty about analytic judgments, inputs, or projections affect findings. Report the effect of choice of discount rate and time horizon, if applicable.	Not applicable

Topic	No.	Item	Location where item is reported
**Effect of engagement with patients and others affected by the study**	25	Report on any difference patient/service recipient, general public, community, or stakeholder involvement made to the approach or findings of the study	Results, starting at line 205
**Discussion**			
**Study findings, limitations, generalisability, and current knowledge**	26	Report key findings, limitations, ethical or equity considerations not captured, and how these could affect patients, policy, or practice.	Discussion, starting at line 225
**Other relevant information**			
**Source of funding**	27	Describe how the study was funded and any role of the funder in the identification, design, conduct, and reporting of the analysis	Line 411
**Conflicts of interest**	28	Report authors conflicts of interest according to journal or International Committee of Medical Journal Editors requirements.	Line 406

From: Husereau D, Drummond M, Augustovski F, et al. Consolidated Health Economic Evaluation Reporting Standards 2022 (CHEERS 2022) Explanation and Elaboration: A Report of the ISPOR CHEERS II Good Practices Task Force. Value Health 2022;25. doi:10.1016/j.jval.2021.10.008

For peer review only - http://bmjopen.bmj.com/site/about/guidelines.xhtml

BMJ Open

The cost of SARS-CoV-2 self-test distribution programs by different modalities, a microcosting study in five countries: Brazil, Georgia, Malaysia, Ethiopia, and the Philippines

Journal:	*BMJ Open*
Manuscript ID	bmjopen-2023-078852.R1
Article Type:	Original research
Date Submitted by the Author:	19-Feb-2024

Complete List of Authors:	Hansen, Megan; Amsterdam UMC Location AMC, Medical Microbiology; Boston University School of Public Health, Global Health<br>Lekodeba, Nkgomeleng; Health Economics and Epidemiology Research Office<br>Chevalier, Joshua; Amsterdam UMC Locatie AMC, Medical Microbiology; Boston University School of Public Health, Global Health<br>Ockhuisen, Tom; Amsterdam UMC Location AMC, Medical Microbiology<br>del Rey-Puech, Paula; Foundation for Innovative New Diagnostics<br>Marban-Castro, Elena; Foundation for Innovative New Diagnostics<br>Martínez-Pérez, Guillermo; Foundation for Innovative New Diagnostics<br>Shilton, Sonjelle; Foundation for Innovative New Diagnostics<br>Radzi Abu Hassan, Muhammad; Hospital Sultanah Bahiyah, Clinical Research Center<br>Getia, Vladimir; National Center for Disease Control and Public Health, Lugar Research Center<br>Weinert-Mizuschima, Catiuscia; LIFEMED<br>Tenorio Bezerra, Maria Isabelle; Secretaria Municipal de Saúde<br>Chala, Lensa; KNCV Tuberculosis Foundation<br>Leong, Robert; KNCV Tuberculosis Foundation<br>Peregino, Remilekun; The Aurum Institute for Health Research<br>Keller, Sara; KNCV Tuberculosis Foundation<br>Spruijt, Ineke; KNCV Tuberculosis Foundation<br>Johnson, Cheryl; World Health Organization<br>Girdwood, Sarah; Foundation for Innovative New Diagnostics; Health Economics and Epidemiology Research Office<br>Nichols, Brooke E.; Amsterdam UMC Location AMC, Medical Microbiology; Foundation for Innovative New Diagnostics

**Primary Subject Heading**:	Health economics
Secondary Subject Heading:	Diagnostics, Global health, Public health, Respiratory medicine
Keywords:	COVID-19, Health economics < HEALTH SERVICES ADMINISTRATION & MANAGEMENT, Public health < INFECTIOUS DISEASES, Respiratory infections < THORACIC MEDICINE

SCHOLARONE™
Manuscripts

I, the Submitting Author has the right to grant and does grant on behalf of all authors of the Work (as defined in the below author licence), an exclusive licence and/or a non-exclusive licence for contributions from authors who are: i) UK Crown employees; ii) where BMJ has agreed a CC-BY licence shall apply, and/or iii) in accordance with the terms applicable for US Federal Government officers or employees acting as part of their official duties; on a worldwide, perpetual, irrevocable, royalty-free basis to BMJ Publishing Group Ltd ("BMJ") its licensees and where the relevant Journal is co-owned by BMJ to the co-owners of the Journal, to publish the Work in this journal and any other BMJ products and to exploit all rights, as set out in our licence.

The Submitting Author accepts and understands that any supply made under these terms is made by BMJ to the Submitting Author unless you are acting as an employee on behalf of your employer or a postgraduate student of an affiliated institution which is paying any applicable article publishing charge ("APC") for Open Access articles. Where the Submitting Author wishes to make the Work available on an Open Access basis (and intends to pay the relevant APC), the terms of reuse of such Open Access shall be governed by a Creative Commons licence – details of these licences and which Creative Commons licence will apply to this Work are set out in our licence referred to above.

Other than as permitted in any relevant BMJ Author's Self Archiving Policies, I confirm this Work has not been accepted for publication elsewhere, is not being considered for publication elsewhere and does not duplicate material already published. I confirm all authors consent to publication of this Work and authorise the granting of this licence.

For peer review only

The cost of SARS-CoV-2 self-test distribution programs by different modalities, a microcosting study in five countries: Brazil, Georgia, Malaysia, Ethiopia, and the Philippines

Authors: Megan A. Hansen[1,2], Nkgomeleng Lekodeba[3], Joshua M. Chevalier[1,2], Tom Ockhuisen[1], Paula del Rey-Puech[4], Elena Marban-Castro[4], Guillermo Zohar Martinez-Perez[4], Sonjelle Shilton[4], Muhammad Radzi Abu Hassan[5], Vladimir Getia[6], Catiuscia Weinert-Mizuschima[7], Maria Isabelle Tenorio Bezerra[8], Lensa Chala[9], Robert Leong[10], Remilekun Roland Peregino[11], Sara Keller[12], Ineke Spruijt[12], Cheryl Johnson[13], Sarah J. Girdwood[3,4*], Brooke E. Nichols[1,3,4*]

Affiliations:

1. Department of Medical Microbiology, Amsterdam University Medical Center, Amsterdam, The Netherlands
2. Department of Global Health, Boston University School of Public Health, Boston, Massachusetts, United States of America
3. Health Economics and Epidemiology Research Office, Faculty of Health Sciences, University of the Witwatersrand, Johannesburg, South Africa
4. FIND, Geneva, Switzerland
5. Clinical Research Center, Hospital Sultanah Bahiyah, Alor Setar, Malaysia
6. Lugar Research Center, National Centers for Disease Control and Public Health, Tbilisi, Georgia
7. LIFEMED, Pelotas, Rio Grande do Sul, Brazil
8. Secretaria Municipal de Saúde, Afogados da Ingazeira, Pernambuco, Brazil
9. KNCV Tuberculosis Foundation, Addis Ababa, Ethiopia
10. KNCV Tuberculosis Foundation, Manilla, The Philippines
11. Aurum Institute, Accra, Ghana
12. KNCV Tuberculosis Foundation, The Hague, The Netherlands
13. World Health Organization (WHO), Geneva, Switzerland

Correspondence: Brooke E Nichols, brooke.nichols@finddx.org *shared contribution

For peer review only

For peer review only - http://bmjopen.bmj.com/site/about/guidelines.xhtml

Abstract

Objective:

Diagnostic testing is an important tool to combat the COVID-19 pandemic, yet access to and uptake of testing varies widely three years into the pandemic. The World Health Organization recommends the use of COVID-19 self-testing as an option to help expand testing access. We aimed to calculate the cost of providing COVID-19 self-testing across countries and distribution modalities.

Design:

We estimated economic costs from the provider-perspective to calculate the total cost and the cost per self-test kit distributed for three scenarios that differed by costing period (pilot, annual), the number of tests distributed (actual, planned, scaled assuming an epidemic peak), and self-test kit costs (pilot purchase price, 50% reduction).

Setting:

We utilized data collected between August and December 2022 in Brazil, Georgia, Malaysia, Ethiopia, and the Philippines from pilot implementation studies designed to provide COVID-19 self-tests in a variety of settings – namely workplace and healthcare facilities.

Results:

Across all five countries, 173,000 kits were distributed during pilot implementation with the cost/test distributed ranging from \$2.44–\$12.78. The cost/self-test kit distributed was lowest in the scenario that assumed implementation over a longer period (year), with higher test demand (peak), and a test-kit price reduction of 50% (\$1.04–\$3.07). Across all countries and scenarios, test procurement occupied the greatest proportion of costs: 58%–87% for countries with off-site self-testing (outside the workplace, e.g., home), 15%–50% for countries with on-site self-testing (at the workplace). Staffing was the next key cost-driver, particularly for distribution modalities that had on-site self-testing (29%–35%) versus off-site self-testing (7%–27%).

Conclusions:

Our results indicate that it is likely to cost between \$2.44–\$12.78 per test to distribute COVID-19 self-tests across common settings in five heterogeneous countries. Cost-effectiveness analyses using these results will allow policymakers to make informed decisions on optimally scaling-up COVID-19 self-test distribution programs across diverse settings and evolving needs.

For peer review only - http://bmjopen.bmj.com/site/about/guidelines.xhtml

Strengths and limitations of this study

- Strength: This research fills gaps in the lack of existing data to help understand the cost of provision and use of COVID-19 self-tests, particularly in lower middle-income countries (LMICs) and middle-income countries (MICs).
- Strength: The results from this study provide a wide range of costing evidence, in a variety of settings and modalities, to governments making decisions regarding the adoption and scale-up of self-test distribution programs.
- Limitation: This study is not a time in motion analysis to assess the time spent by each staff cadre on pilot-related activities; therefore, our estimates on the average time spent by staff on pilot activities per month may be overestimated.
- Limitation: The pilots in Brazil and Malaysia were ongoing during data collection, requiring the extrapolation of figures and use of budgeted expenditure instead of actual expenditure.

For peer review only

For peer review only - http://bmjopen.bmj.com/site/about/guidelines.xhtml

Introduction

As of July 2023, the COVID-19 pandemic has led to nearly 768 million cases and 6.9 million deaths worldwide [1]. While testing has been acknowledged as a critical tool to combat the spread of SARS-CoV-2, access to testing remains varied nearly three years into the pandemic. In the early stages of the COVID-19 pandemic, polymerase chain reaction (PCR) tests were the preferred method for the diagnosis of SARS-CoV-2. However, as more cases emerged, and transmission became widespread, low-cost ($1.50 per test) antigen-based rapid diagnostic tests have increasingly been utilized to expand access to and uptake of testing [2].

Despite the benefits, many programs found limiting the use of Ag-RDTs (antigen rapid diagnostic tests) to professional administration and use still missed many people in need of testing services [3]. The use of self-testing for Ag-RDTs, where individuals perform and interpret their own diagnostic test, is a mechanism to relieve this human resource constraint and rapidly expand potential testing capacity. These more affordable self-tests also allow individuals to test themselves, promptly learn their results, and take actions that could improve individual health outcomes and/or prevent onward transmission. For these reasons, COVID-19 self-tests have become increasingly popular in high-income countries (HICs) and were recommended by the World Health Organization (WHO) in 2022 [1].

Distribution of self-test kits would allow for the scale-up of COVID-19 testing in limited-resource settings, where regular screening through provider-initiated and/or laboratory-based platforms, such as PCR tests, is constrained. While there are some studies on the use of Ag-RDT self-tests in schools, workplaces, and within the broader community, these studies focus primarily on the usability or accuracy in comparison to provider-offered testing [4–7]. There is limited research on the relative programmatic costs for the implementation and distribution of COVID-19 self-tests through different modalities, especially in low-resource settings or among communities with restricted access to conventional COVID-19 testing, where self-testing may be of most benefit [8].

Most of the empirical evidence on the cost of self-testing programs originates from the Human Immunodeficiency Virus (HIV) self-testing literature [6,9,10], and more recently from Hepatitis C Virus (HCV) self-testing [11]. Studies have demonstrated the relative cost of different methods for rollout of HIV self-testing and found, broadly, that there are multiple ways of successfully distributing HIV self-testing, depending on the budget and desired outcome. For HIV self-testing, expansion of testing beyond primary health care clinics yielded a net increase in life years saved across all distribution models [9]. Workplace

For peer review only - http://bmjopen.bmj.com/site/about/guidelines.xhtml

testing was found to be a very cost-effective modality for HIV self-test distribution. It may be plausible that self-testing for COVID-19 is similarly cost-effective in the workplace.

The HIV literature gives us insights into relative differences in cost of implementation of self-test distribution modalities, as well as which modalities are acceptable, appropriate, and perceived to be convenient for a variety of populations. However, there are differences in modalities and frequency of testing required for HIV or HCV compared to a respiratory virus such as COVID-19. Given these key differences, more evidence is needed to understand the costs related to delivering COVID-19 self-testing through different modalities for future planning – for potential future COVID-19 waves, for other seasonal viruses such as influenza, and possibly for future outbreaks caused by emerging diseases.

Despite the recently popular wide use of COVID-19 self-testing in countries with available public health resources, there has been a paucity of data generated to understand the cost of provision, particularly in lower middle-income countries (LMICs) and middle-income countries (MICs). We aimed to determine the cost per COVID-19 self-test allocated by distribution modality as well as the cost per COVID-19 self-test performed. The overarching aim was to provide costing evidence to governments making decisions regarding the adoption and scale-up of self-test distribution programs to monitor and effectively improve case detection of COVID-19.

Methods

Overview

COVID-19 self-testing pilot studies were implemented in three upper middle-income countries (UMICs): Brazil, Georgia, and Malaysia, as well as one LMIC (the Philippines) and one low-income country (LIC) (Ethiopia). These pilot implementation studies were designed to provide COVID-19 self-tests to employees, their household members, and clinic clients in a variety of different settings – ranging from healthcare settings, to workplaces, to schools (Table 1). The goal of the pilot studies was to assess and continuously improve COVID-19 self-testing distribution strategies, the feasibility of the implementation, and participants' acceptability – as an appropriate intervention to increase uptake of testing and detection of cases of COVID-19 in the target populations [7]. Additionally, these pilot studies aimed to calculate the cost of providing COVID-19 self-testing via several distribution modalities in different countries.

For peer review only - http://bmjopen.bmj.com/site/about/guidelines.xhtml

140 ***Study setting and scope***

141 This is a multi-site, prospective cohort study across five countries and multiple workplaces and health 142 facilities (Table 1).

143

144 **Table 1.** Description of distribution models. *Off-site testing: refers to self-testing outside the workplace 145 (e.g., at home) On-site testing: refers to self-testing at the workplace.

Country	Distribution Modality*	Pilot Implementation Time Period	Potential participants and channel	Secondary distribution to household members	Description
Brazil (Pelotas, Rio Grande do Sul)	Staff at a workplace of a private medical supply factory for primarily off-site testing	August 2022 – February 2023	Primary distribution to 358 employees	Yes	For the first month, four self-tests were distributed per participant to perform weekly testing. Afterwards, up to six self-test kits were distributed to staff members per month, with recommended use of one per week or at the onset of any COVID-19 symptoms. Results were recorded through a digital platform used at the workplace (mobile application) and participants were expected to seek confirmatory testing, following national guidelines.
Brazil (Afogados da Ingazeira, Pernambuco)	Professionals at sites in both the public healthcare sector (20) and the public education sector (14) for primarily off-site testing	October 2022 – March 2023	Primary distribution to 344 employees working in healthcare and 855 employees working in the education sector (up to 1,200 total participants)	Yes	During the first month employees were encouraged to perform one self-test per week regardless of if COVID-19 symptoms were present or not (resulting in approximately four tests per month), and an additional self-test is recommended if symptoms arise throughout the week. Afterwards, employees were encouraged to self-test in case of symptoms or contact with a positive case. Results were recorded through a survey link sent via Google Forms and participants were expected to seek confirmatory testing, following national guidelines.
Georgia (Tbilisi, Kutaisi and Svaneti	Staff at schools in a remote mountainous region (24),	June 2022 – December 2022	Primary distribution to 2,066 employees	Yes	Primary self-test kit distribution to staff members at pilot sites for weekly testing, as part of the mandatory national testing program, and testing in case of symptoms or contact with a

For peer review only - http://bmjopen.bmj.com/site/about/guidelines.xhtml

region)	nursing homes (2) and healthcare centers (hospitals (2) and clinics (1)) for primarily off-site testing				positive case, as well as secondary self-test kit distribution to staff members' household members for use in the case of developing symptoms or having a close contact with a positive COVID-19 case. Results were reported to designated staff right after use to be entered in the national database. Positive staff members were linked to care.
Malaysia (Alor Setar, Sungai Petani, and wider state of Kedah)	Staff of private manufacturing companies (4) for primarily off-site self-testing	November 2022 to April 2023	Primary distribution to up to 2,000 employees	Yes	Initial distribution of two self-test kits per staff and household member for self-testing in case of symptomatic/contact/other circumstances, following national guidelines. Kits were refilled on an as-needed basis. Participants received training and information materials on-site prior to test distribution. Participants were asked to report test-use and results by Google Form.
Ethiopia (Addis Ababa and regional states of Amhara and Oromia)	Clients at clinics for on-site self-testing (2)	September 2022 – November 2022	Primary distribution to clients eligible for self-testing when visiting the clinics (total tests available 1250)	No	Clients arrived at clinic to seek health care and were screened for eligibility for partaking in the pilot. If the client was eligible, a self-test kit was provided for on-site self-testing. Clients performed test, interpret, and read results while supervised by nurses. If the self-test was positive, the client was sent for confirmatory testing.
Philippines (Biñan, Los Baños, and wider province of Laguna)	Staff at workplace clinics for on-site testing (7)	September to December 2022	Primary distribution to workers working at partner clinics. (total tests available 500/site/month)	No	Distribution of self-test kits at partner workplace, with on-site assigned facility to be its clinic. Distribution agents demonstrated self-test kit use to groups. Participants were tested on-site by a provider under supervision of clinic staff.

Costing inputs and data collection

We estimated economic costs from the provider perspective using data collected between August and November 2022 in Brazil, Georgia, Malaysia, Ethiopia, and the Philippines. All the costs were either collected in United States Dollars (USD) or the countries' local currency and converted to 2022 USD using the average exchange rate for the period January to October 2022 sourced from the country's

For peer review only - http://bmjopen.bmj.com/site/about/guidelines.xhtml

central/national banks [12–17].

The costing approach used was a combination of expenditure analysis in estimating financial costs and bottom-up costing to identify any items not included in pilot expenditure records [18]. Cost data was collected across all countries and modalities using a standardized Excel data collection tool that categorized the costs as follows: training, test procurement, distribution and storage, staff costs (management and service delivery), data reporting, supplies and consumables, and communication (Table 2; details for each country and modality in Appendix A1-A6). Costs in Brazil, Georgia, and Malaysia were collected while our team travelled to the sites and investigated financial records. Costs in Ethiopia and the Philippines were reported by our in-country research partners.

Shared costs were allocated to each distribution modality using the proportion of tests distributed and the proportion of staff for costs driven by this (for example, training costs). Staff salaries were obtained from the in-country implementing partners and allocated by the proportion of time spent on COVID-19 self-test distribution as estimated by in-country implementing partners. Where appropriate, staff salaries for the equivalent staff cadre within the Ministry of Health or government were used in order to better align with routine implementation assumptions in the future.

Table 2. Cost categories and description

**Cost category**	**Description & explanation**
Test procurement	• Cost of COVID-19 self-test kit unit<br>• Shipping, customs clearance, duties, taxes
Test distribution	• Transportation cost (e.g., fuel) for the transportation of COVID-19 self-tests to sites (including transport to central storage location if relevant)
Test storage	• Purchase or rental of storage space for COVID-19 self-test, such as a warehouse or storage unit<br>• Purchase of any necessary accompanying supplies used for storage (e.g., shelves and boxes)
Training	• Training sessions of staff that are members of the team that are dedicated to the COVID-19 self-test activities<br>• Training sessions for the participants on how and when to self-test, how to report the results, and where to find more information about self-testing<br>• Design and printing of training materials<br>• Venue, catering, supplies, and transport costs associated with training

For peer review only - http://bmjopen.bmj.com/site/about/guidelines.xhtml

Communication	● Designing and ordering advertisement posters and stickers<br>● Designing and printing information materials, such as letters and pamphlets<br>● Designing and distributing digital communication materials (e.g., instructional videos, digital infographics)<br>● Any additional communication activities that may accrue cost, such as awareness trainings<br>● Any internet or mobile associated communication costs
Staff: management	● Cost of staff time spent on activities to manage the COVID-19 self-testing campaign<br>● Cost of time spent on project implementation and support (including any consultant's time where appropriate)
Staff: service delivery	● This includes the salary of the staff, and the average proportion of their time spent dedicated to COVID-19 self-test operations:<br>● Activities include staff time spent on:<br>○ Collecting COVID-19 self-tests to bring to appropriate testing facility (e.g., workplace, clinic)<br>○ Storing and organizing COVID-19 self-tests<br>○ Enrollment sessions for initiation of COVID-19 self-test distribution to participants<br>○ Assisting or administering the COVID-19 self-test to participants<br>○ Training participants on how to use the COVID-19 self-test, including information and sensitization activities, answering questions during the pilot
Data and reporting	● Development and use of a platform where results are documented and reported<br>● Record keeping after COVID-19 self-tests are distributed, maintaining registers, data compilation and entry<br>● Analyzing reported records and completing reports<br>● Technical working group or other meetings to discuss and report on data
Supplies and consumables	● Any infection, prevention and control materials used (masks, hand sanitizers, gloves, alcohol, biohazard bags) for on-site testing or training<br>● Any office supplies used for recording results etc. (pens, paper)

Scenario analysis

We calculated the total cost and the cost per self-test kit distributed for three main scenarios, shown in Table 3, that differed depending on the costing period used (actual pilot period compared to a theoretical one year implementation period), the number of tests distributed (actual, planned, scaled assuming an epidemic peak), the price of the self-test kit (pilot price, and a reduction of 50%); and a final scenario considering scaled-up peak implementation with the exclusion of staffing costs assuming that self-test kit distribution would be integrated with other routine services and the staff costs absorbed by the respective provider (workplace, healthcare facility). We included a 50% reduction in the purchase price of the tests for Scenarios 2 and 3 with the expectation that with possible increased testing volumes, that the cost of the test

For peer review only

would reduce, as seen with other diagnostic tests.

Table 3. Three different implementation scenarios. Three different implementation scenarios with different sub-scenarios and other variables impacting the cost analyses

Scenario	Scenario description	Period	Number of tests	Cost
1	Pilot costing	Pilot period (preparation time + implementation)	Actual distribution as of 30th November 2022 extrapolated for the remaining period of the pilot	Pilot test kit cost
2	Routine implementation on annual basis	Annual (12 months)	Planned distribution extrapolated to 12 months	50% reduction in the price of the test kit
3	Scaled-up peak implementation*	Annual (12 months)	Doubling of planned annual test distribution to accommodate an epidemic peak period (increased testing for the same number of beneficiaries)	a) 50% reduction in the price of the test kit<br>b) 50% reduction in the price of the test kit with no data platform costs and no staff costs**

*Assumed that staff did not have to increase to facilitate a doubling of tests distributed

**Assumed that staff and data/reporting costs would be absorbed by the respective provider (workplaces, or health facilities) and self-test kit delivery integrated into other activities and existing health data platforms

Ethics

The Georgia study was granted ethical approval by the National Center for Disease Control (NCDC) and Public Health Institutional Review Board in Tbilisi, Georgia (Ref.: # 2022-049, May 24, 2022). The Malaysia study was ethically approved by the Medical Research and Ethics Committee (MREC), Ministry of Health Malaysia in Putrajaya, Malaysia (Ref.: NMRR ID-22-02021-UCR, Oct 26, 2022). The pilot in Ethiopia was granted ethical approval by the Ethiopian Public Health Institute Institutional Review Board (EPHI-IRB) in Addis Ababa, Ethiopia (Ref.: EPHI-IRB-421 2022, Aug 19, 2022). The study in the Philippines was approved by Saint Cabrini Medical Center – Asian Eye Institute Ethics Review Committee (SCMC–AIE ERC) in Manila, the Philippines (Ref.: QR-ERC-002/05/00/12032020, Oct 19, 2022).

Based on the provisions of Chapter IX, items I and IX, of Resolution 674 of 2022, approval from the

For peer review only

For peer review only - http://bmjopen.bmj.com/site/about/guidelines.xhtml

Research Ethics Committee/National Research Ethics Council (CEP/CONEP System) was waived for the pilot projects in Pelotas and Afogados da Ingazeira, Brazil, as the program only used public domain data, did not identify the participants, and were conducted alongside routine operations at the targeted workplaces. Furthermore, to ensure the safety of individuals enrolled, the program was carried out considering all ethical principles defined in Brazilian legislation on the General Personal Data Protection Law (LGPD) [19]. All welcomed employees voluntarily signed the Free and Informed Consent Form (ICF) after agreeing to participate in the program. The socio-economic evaluation was designed, conducted, and reported in accordance with consolidated criteria for reporting health economic evaluations [20].

Patient and Public Involvement

Participants were involved in the design, conduct, and reporting of the research through inclusion in trainings, enrollment sessions, and reporting of data through various platforms as described in Table 1.

Results

Across all five countries, there were a total of 173,000 test-kits distributed during the pilot period (Table 4). Georgia had the largest pilot program (90,000 tests distributed); and Ethiopia the smallest (158 at the two healthcare facilities costed (this was scaled back from a planned distribution of 1250 kits). The cost per test distributed ranged from $2.44 (Malaysia) to $12.78 (Ethiopia). Despite having the lowest price per self-test kit purchased ($1.00), Ethiopia had the highest cost per test distributed ($12.78). This was due in part to low test volumes and higher resource intensity (i.e., staff time) associated with on-site testing at a healthcare facility. Malaysia, in comparison, had the lowest cost per test distributed of $2.44, followed by public workplaces in Afogados da Ingazeira, Brazil ($2.82).

The cost per self-test kit distributed across all countries decreases with a routine implementation period of a year (Scenario 2 and 3) and with an increase in the scale of the program (Scenario 3) (Table 4). The cost per self-test kit distributed, while still considering staff and reporting costs, is lowest in Scenario 3 with higher test demand (during peaks of incidence) assumed, and a test kit price reduction of 50%: ranging from $1.04 in Malaysia to $3.37 in Brazil (Pelotas). The total cost is however high for both Scenario 2 and 3 due to the high procurement costs associated with procuring more tests as well as the longer period of implementation, up to $1.7M for the largest program in Georgia. When assuming that self-test kit distribution would become a routinely integrated service with a reduced purchase price, and assuming zero staffing and data/reporting costs, the cost per test for Scenario 3 ranged from $0.86 in Malaysia to $2.55 in Brazil (Pelotas). However, if data and reporting costs were not absorbed by existing health data platforms,

For peer review only - http://bmjopen.bmj.com/site/about/guidelines.xhtml

data and reporting would only increase the cost per self-test by \$0.00–\$0.12 in Malaysia and Brazil (Pelotas), respectively.

Figure 1 summarizes the proportion of total costs that each cost category contributes to across scenarios and countries. Across all countries and scenarios, test procurement (the price of the self-test kits themselves including the costs shipping etc.) contributes the largest proportion and becomes even more important at greater scale (Scenario 3). Test procurement costs range from 58%–87% for countries with off-site testing (Georgia, Malaysia, Brazil), and 15% to 50% for countries with on-site testing (Ethiopia and the Philippines). Staff costs was the next key cost driver, particularly for distribution modalities that had on-site testing (29%–35%) versus distribution modalities with off-site testing (7%–27%). Service delivery staffing costs (staff directly involved in the distribution of the self-test kits) ranged from as little as 3% in Malaysia (Scenario 3) to 26% in Ethiopia (Scenario 3). In the Philippines, Malaysia and both pilots in Brazil, staff management costs were the second largest cost driver ranging from 6% to 25% of total costs but decreasing with the scale of the program. This is particularly notable in the case of Ethiopia, which saw a sharp drop-off in the contribution of staff management costs when moving from a pilot program to more routine implementation as fixed management costs are spread across a higher number of tests.

For peer review only - http://bmjopen.bmj.com/site/about/guidelines.xhtml

Table 4. Program characteristics and all-inclusive cost per test for each country during the pilot (Scenario 1) and annual routine periods of implementation (Scenario 2 and 3). An extended version of this table can be found in the Appendix A7.

	**Scenario 1 – pilot implementation**		**Scenario 2 – routine implementation**			**Scenario 3 – scaled peak implementation†**		
**Country** & distribution method	**Total tests distributed** with purchase price of self-test kit	**Cost per test:** all-inclusive	**# tests distributed**	**Cost per test:** using current self-test kit price	**Cost per test:** using reduced self-test kit price	**Cost per test:** using current self-test kit price	**Cost per test:** using reduced self-test kit price	**Cost per test:** with integrated staff, data, & reporting
**Brazil (Pelotas)** -Primarily off-site -Unassisted	**10,000** @ $4.50	$7.38	**20,000**	$6.54	$4.29	$5.62	$3.37	$2.55
**Brazil (Afogados)** -Primarily off-site -Unassisted	**26,000** @ $2.00	$2.82	**52,000**	$2.59	$1.59	$2.30	$1.30	$1.05
**Georgia** -Off-site -Unassisted	**90,000** @ $2.00	$4.44	**300,000**	$3.34	$2.34	$2.95	$1.95	$1.61
**Malaysia** -Off-site -Unassisted	**45,000** @ $1.55	$2.44	**135,000**	$2.06	$1.28	$1.82	$1.04	$0.86
**Ethiopia** -On-site -Provider assisted	**158** @ $1.00	$12.78	**5,040**	$4.22	$3.72	$3.80	$3.30	$1.97
**Philippines** -On-Site -Unassisted	**2,000** @ $1.32	$4.99	**6,000**	$4.51	$3.85	$2.92	$2.26	$0.95

†Twice as many tests are distributed in Scenario 3 as in Scenario 2 (as also described in Table 3).

Figure 1. Cost drivers and the cost per test distributed by country and scenario at current self-test kit procurement prices.

For peer review only - http://bmjopen.bmj.com/site/about/guidelines.xhtml

Discussion

We aimed to determine the cost per COVID-19 self-test allocated by distribution modality as well as the cost per COVID-19 self-test performed. Across all five countries analyzed in this study, there were a total of 173,000 kits distributed with the cost per test distributed ranging from $2.44–$12.78. The cost per self-test kit distributed is lowest in the scenario that assumed implementation over a longer period (one year), with higher test demand (peak), and a test kit price reduction of 50% ($1.04–$3.07).

COVID-19 self-tests are generally cost-saving compared to PCR-assays and professional use Ag-RDTs. The average cost of an automated PCR-assay is at least $10.00, excluding the cost of sample collection or result delivery [2]. In comparison, the cost of a COVID-19 self-test in the pilot studies ranged from $1.00 to $4.50 making them a more affordable option for expanding access to testing and thereby increasing detection of COVID-19 cases and enacting recommended preventative measures [2]. Despite similarities across countries in terms of income categorization (three countries are categorized as MICs) as well as distribution modality (five out of the six were workplace distribution models), the cost of *distributing* COVID-19 self-tests varied substantially. This variability in cost can be attributed to the type of COVID-19 self-test that were used (as shown above), the amount of self-tests that were procured, and the modality of distribution or pilot implementation methodology. We found that across countries and scenarios, test procurement (the price of the self-test kit and shipping) constitutes the highest proportion of the total costs of distribution: 58% to 87% for countries with off-site testing and 15% to 50% for countries with on-site testing. Whilst this might suggest that country governments have little flexibility in influencing the overall cost of distributing COVID-19 self-tests outside of procurement negotiations, there are still substantial costs related to the delivery of COVID-19 tests and as such potential for cost reduction, particularly through the design of the distribution modality itself (for example, on-site versus off-site testing). Additionally, the substantial cost of test procurement and shipping may indicate that purchasing COVID-19 self-tests from regional manufacturers or better supply management and coordination may help reduce costs.

We can see that tests that were performed on-site resulted in a greater unit cost per self-test compared to tests that were performed off-site. For example, the on-site pilot implemented in healthcare facilities in Ethiopia ($12.78), required a significantly greater amount of staff-time and consumables when compared to the off-site pilot implemented in Malaysia ($2.44). We would expect to see further deviation in the cost of distribution of self-tests in both HICs and LMICs as donor funding and access to Ag-RDTs differs in settings with high and low resources respectively. Additionally, we can see that the cost to distribute COVID-19 self-tests can be expected to differ during routine program implementation rather than pilot

For peer review only - http://bmjopen.bmj.com/site/about/guidelines.xhtml

implementation. We attempted to estimate how start-up costs and pilot staff planning and management costs dedicated to the self-test program would be reduced during routine implementation (Scenarios 2 and 3). This fluctuation in cost can be described as the "U-shape phenomenon"; the cost per test distributed is initially high but decreases as the program scales-up distribution since fixed start-up (data systems, planning and communication design) and once-off costs (training) are spread over a greater number of tests. Further, we showed how this cost is reduced when the distribution of self-test kits is completely integrated into routine operations and the staffing costs are incrementally small and absorbed by the provider. However, at a certain level of scale, the cost per test will increase again as diseconomies of scale initiate as well as when additional staff and training is required to facilitate the distribution of more and more tests.

Prior to this study, little was known about the cost to implement COVID-19 self-testing programs, however, self-test distribution has been offered via different modalities for various infectious diseases, including HIV, in which we can draw some comparisons. *Sande et al.* found that the key cost drivers of offering HIV self-test kits at public health facilities across four sub-Saharan African countries were personnel and the cost per test kit [6]. This was largely true across distribution modalities and countries for COVID-19 self-testing, as test procurement comprised the largest contribution to total costs, followed by staff management and service delivery categories, even though the cost per test procured differed by country. In the analysis by *Sande et al.*, they observed economies of scale across sites, as the cost per HIV self-test kit distributed was lower among sites that distributed more test kits, with the cost ranging from $4.27–13.42 [6]. We observed a similar trend in our analysis when scaling up the number of COVID-19 self-test kits distributed, assuming staff costs remain consistent, and start-up related costs begin to taper off, with increased distribution. In another analysis by *Matsimela et al.* that looked at HIV self-test kit distribution across 11 modalities in South Africa, including workplaces, they observed economies of scale related to test kit distribution volume. Test kit cost and personnel were indeed the greatest cost drivers in the workplace modalities in that analysis, with the cost to distribute an HIV self-test in the workplace ranging from $5.30–6.29 [10]. While we cannot directly compare the cost to distribute an HIV self-test kit to a COVID-19 self-test kit due to the inherently different nature of the two viruses' epidemiology (COVID-19 can require more frequent repeat testing), as well as the nature and stigmatization of diagnosing HIV, we do see a similar range in the cost per COVID-19 self-test kit distributed from $2.44–12.78. Our analysis provides an overview of the potential overall costs to offer COVID-19 self-testing programs.

To our knowledge, this study is the first to estimate the costs of COVID-19 self-test distribution across different countries and settings. We determined the costs based on the best available evidence from pilot studies conducted in five heterogeneous countries across predominately workplace distribution modalities.

For peer review only - http://bmjopen.bmj.com/site/about/guidelines.xhtml

There are however several limitations. Firstly, whilst we tried to exclude study-specific costs, there might be protocol-induced higher resource-use costs – which may not be observed at scale-up (including higher uptake of testing and reporting due to pilot-specific follow-up). This might result in higher total costs, but also more tests performed potentially resulting in a net effect on the cost per test. Additionally, we did not conduct a time in motion analysis to assess the time spent by each staff cadre on pilot-related activities. As such, it is probable that our estimates on the average time spent by staff on pilot activities per month are overestimated. It is likely that the time spent on test kit distribution will decrease after the starting months, and especially with routine implementation. We tried to adjust for this by excluding all staff costs under Scenario 3 to provide a lower bound of what this cost might be, especially with a 50% reduction in the purchase price of the self-tests. Also, in Scenario 3 we have assumed that there would be no need for additional staff or staff time if the number of tests distributed were to double. However, since it is possible that we overestimated the average time spent by staff on pilot activities per month, we felt that this assumption was reasonable. In some countries (Brazil and Malaysia), the pilot was ongoing, and we had to extrapolate figures (actual COVID-19 test distribution), as well as use budgeted expenditure instead of actual expenditure. Across countries, actual distribution was lower than planned distribution due to low COVID-19 prevalence. The stage of the epidemic would affect the uptake of self-tests and subsequently the cost per test. To account for this, Scenario 3 assessed a doubling of testing demand as a result of an assumed peak in the epidemic to approximate high demand or uptake phases. Lastly, outside of the workplace distribution model, the only other modality piloted was the on-site testing at healthcare facilities in Ethiopia. As such, we were unable to understand the relative difference in implementation costs across modalities limiting the generalizability of these costs to other distribution modalities.

Conclusion

Our results indicate that it is likely to cost between $2.44 and $12.78 per test to distribute COVID-19 self-tests across common settings in five heterogeneous countries with differing economical characteristics, health systems governance, and epidemiological and demographic profiles. Additional costing and cost-effectiveness analyses based on these results will allow policy makers to make informed decisions on optimal approaches to scaling up COVID-19 self-test distribution programs across settings and countries as epidemiology and testing needs continue to evolve.

For peer review only - http://bmjopen.bmj.com/site/about/guidelines.xhtml

References

1. WHO. Use of SARS-CoV-2 antigen-detection rapid diagnostic tests for COVID-19 self-testing. Interim Guide. 2022; 1–16.

2. The Global Fund. Pooled Procurement Mechanism Reference Pricing: COVID-19 diagnostics 2022. Available: https://pfscm.org/wpcontent/uploads/2021/12/PFSCM_COVID_PCR_Guidance_2021.pdf

3. Han AX, Girdwood S, Khan S, Sacks JA, Toporowski A, Huq N, et al. Strategies for using antigen rapid diagnostic tests to reduce transmission of SARS-CoV-2 in low- and middle-income countries: a mathematical modelling study. medRxiv. [cited 25 Oct 2022] Available: https://www.medrxiv.org/content/10.1101/2022.06.16.22276516v1

4. Hoehl S, Schenk B, Rudych O, Göttig S, Kohmer N, Karaca O, et al. High-Frequency Self-Testing by Schoolteachers for Sars-Cov-2 Using a Rapid Antigen Test: Results of the Safe School Hesse study. Dtsch Arztebl Int. 2021. doi:10.3238/arztebl.m2021.0187

5. Papenburg J, Campbell JR, Caya C, Dion C, Corsini R, Cheng MP, et al. Adequacy of Serial Self-performed SARS-CoV-2 Rapid Antigen Detection Testing for Longitudinal Mass Screening in the Workplace. JAMA Netw Open. 2022;5: e2210559. doi:10.1001/jamanetworkopen.2022.10559

6. Sande LA, Matsimela K, Mwenge L, Mangenah C, Choko AT, D'Elbée M, et al. Costs of integrating HIV self-testing in public health facilities in Malawi, South Africa, Zambia, and Zimbabwe. BMJ Glob Heal. 2021;6: 1–9. doi:10.1136/bmjgh-2021-005191

7. Martínez-Pérez GZ, Shilton S, Saruê M, Cesario H, Banerji A, Batheja D, et al. Self-testing for SARS-CoV-2 in São Paulo, Brazil: results of a population-based values and attitudes survey. BMC Infect Dis. 2022;22: 720. doi:10.1186/s12879-022-07706-7

8. Nwaozuru U, Obiezu-Umeh C, Diallo H, Graham D, Whembolua GL., Bourgeau MJ, Ritchwood TD, et al. Perceptions of COVID-19 self-testing and recommendations for implementation and scale-up among Black/African Americans: implications for the COVID-19 STEP project. BMC Public Heal. 2022; 22: 1. doi:10.1186/s12889-022-13619-6

9. Jamieson L, Johnson LF, Matsimela K, Sande LA, D'Elbée M, Majam M, et al. The cost effectiveness

For peer review only - http://bmjopen.bmj.com/site/about/guidelines.xhtml

For peer review only

and optimal configuration of HIV self-test distribution in South Africa: A model analysis. BMJ Glob Heal. 2021;6: 1–10. doi:10.1136/bmjgh-2021-005598

10. Matsimela K, Sande LA, Mostert C, Majam M, Phiri J, Zishiri V, et al. The cost and intermediary cost-effectiveness of oral HIV self-test kit distribution across 11 distribution models in South Africa. BMJ Glob Heal. 2021;6: 1–10. doi:10.1136/bmjgh-2021-005019

11. Walker JG, Ivanova E, Jamil MS, Prof A, Ong JJ, Easterbrook PP, et al. Cost and cost-effectiveness of Hepatitis C virus self-testing in four settings: an economic evaluation. 2022.

12. National Bank of Ethiopia. Inter-Bank Daily Foreign Exchange Rate - National Bank. [cited 25 Nov 2022]. Available: https://nbe.gov.et/inter-bank-daily-foreign-exchange-rate-in-usd/

13. National Bank of Georgia. Statistics Data: Exchange Rates. [cited 25 Nov 2022]. Available: https://nbg.gov.ge/en/statistics/statistics-data?title=&code=

14. The Central Bank of Brazil. Exchange rates: Quotations and Bulletins. [cited 25 Nov 2022]. Available: https://www.bcb.gov.br/en/financialstability/quotations%0A

15. The Central Bank of Malaysia. Rates and Statistics: Exchange Rates. [cited 25 Nov 2022]. Available: https://www.bnm.gov.my/exchange-rates

16. The Central Bank of the Republic of Philippines. Statistics: Exchange Rate. [cited 25 Nov 2022]. Available: https://www.bsp.gov.ph/SitePages/Statistics/ExchangeRate.aspx

17. Fuel economy in Brazil IEA. Fuel Economy in Brazil. [cited Jan 2023]. Available: https://www.iea.org/articles/fuel-economy-in-brazil

18. Boonstoppel L, Banks C, Moi F, Vaughan K, Ozaltin A, Brenzel L. How to Conduct an Immunization Campaign Costing Study: Methodological Guidance. 2021. Washington, DC: ThinkWell.

19. Conheça os princípios e as bases legais que dão suporte à Lei Geral de Proteção de Dados Pessoais. [cited Nov 2023]. Available: https://www.serpro.gov.br/lgpd/menu/tratamento-dos-dados/principios-da-lgpd

For peer review only - http://bmjopen.bmj.com/site/about/guidelines.xhtml

20. Husereau D, Drummond M, Petrou S, Carswell C, Moher D, Greenberg D, et al. Consolidated Health Economic Evaluation Reporting Standards (CHEERS) statement. 2013. BMJ; 346 :f1049 doi:10.1136/bmj.f1049

For peer review only

Acknowledgements

We would like to thank all the in-country study teams and implementing partners for their assistance with acquiring data: Brazil (Antakly: Gisela Antakly and Joselito Pedrosa and Germsure: Ana Flavia Pires and Cassia Gonçalves, Pelotas: Lifemed, Afogados da Ingazeira: Municipal Health Secretary); Malaysia (FIND: Xiao Hui Sem and Farhana Zulkifli; MOH of Malaysia: Dr Sunita Binti Abdul Rahman and Dr Chan Huan-Keat); Ethiopia (KNCV: Lensa Chala and Israel Mitiku); Philippines (KNCV: Robert Leong); Georgia (FIND: Maia Japaridze and Ia Jikia; National Center for Disease Control); and all sites participating in the pilots' implementation.

Author Contributions

Conceptualization: BEN Data curation: PDRP, EMC, GZMP, LC, RL Formal analysis: MH, NL, JMC, TO, SJG Investigation: MH, NL, JMC, TO, SJG Methodology: MH, NL, JMC, TO, SJG Supervision: SJG, BEN Writing - original draft: MH, NL, JMC, TO, SJG, BEN Writing – review and editing: MH, NL, JMC, TO, PDRP, EMC, GZMP, SS, MRAH, VG, CWM, MITB, LC, RL, RP, SK, IS, CJ, SJG, BEN

Competing Interests

The authors declare no conflict of interest. The funders had no role in the design of the study; in the collection, analyses, or interpretation of data; in the writing of the manuscript; or in the decision to publish the results.

Funding

This costing study was funded by KfW Group, the German state-owned investment and development bank; grant number KfW-TBBU02. The funders played no role in the design of the study; the collection, management, analysis, or interpretation of data; writing of the report; or the decision to submit the report for publication.

Data Availability Statement

Anonymized cost data is available.

Appendix

Detailed costing tables for each distribution modality, indicating costing assumptions and describing costs included.

For peer review only - http://bmjopen.bmj.com/site/about/guidelines.xhtml

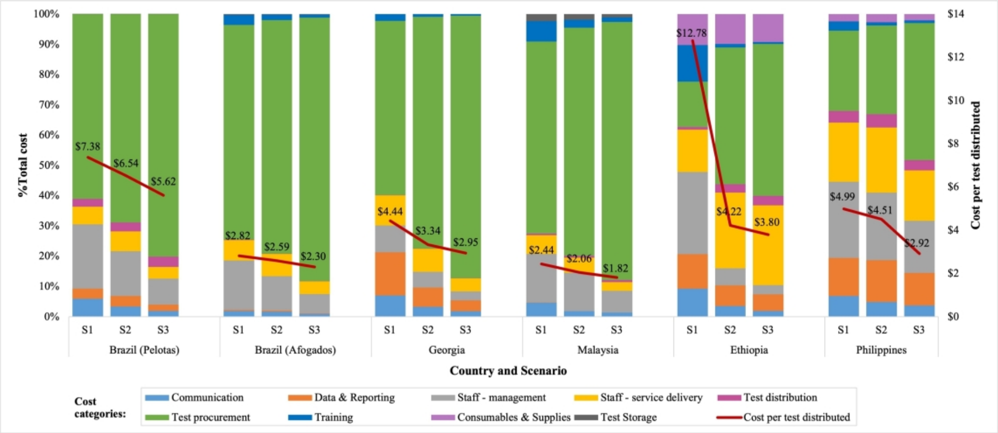

Figure 1. Cost drivers and the cost per test distributed by country and scenario at current self-test kit procurement prices.

283x123mm (300 x 300 DPI)

Supplementary Materials

Appendices of Cost Categories with Descriptions

Table of Contents

Table A1. *Cost categories with descriptions for Pelotas, Brazil* ….………………………..... p. 1

Table A2. *Cost categories with descriptions for Afogados da Ingazeira, Brazil* …………….. p. 2

Table A3. *Cost categories with descriptions for Georgia* …………………..…………………… p. 3

Table A4. *Cost categories with descriptions for Malaysia* ..………………………………….. p. 5

Table A5. *Cost categories with descriptions for Ethiopia* ..…………………………………... p. 6

Table A6. *Cost categories with descriptions for the Philippines* ..…………………..……….. p. 8

Table A7. *Program characteristics and all-inclusive cost per test* …………………………. p. 10

Table A8. *CHEERS Checklist* ……………………………………………………………….. p. 11

For peer review only - http://bmjopen.bmj.com/site/about/guidelines.xhtml

For peer review only

Table A1. Cost categories with descriptions for Pelotas, Brazil. For every cost category, all costs included, and any assumptions made are listed. All costs are reported in 2022 USD.

Cost category	Description & explanation
Test procurement	• GeneFinder COVID-19 Ag Self-Test from Osang Healthcare (South Korea)<br>• Self-test unit price (including shipping/importation, taxes, duties, custom clearance): $4.50<br>• 10,000 self-tests procured
Test distribution	• Single shipment from Sao Paulo, Brazil to the workplace storage facility in Pelotas, Brazil at a cost of $0.19 per test.
Test storage	• Tests were stored on-site at the workplace and incurred no additional costs for storage space.<br>• Costs included were employee time required to rearrange storage facility contents to accommodate self-test shipment (2 x staff, 2 hours each x salary per hour).
Training	• Training of pilot beneficiaries on program and proper self-test use.<br>• Training costs were directly related to staff time/salary and are included in staff-management and staff-service delivery cost categories.
Communication	• All communication materials were digital and included posters, banners, and videos to promote COVID-19 self-testing to beneficiaries.<br>• Costs included under communication were for the design of materials (hourly cost of designer and consultant: 80 hours x $40/hour and 60 hours x $20/hour).
Staff: Management	• Project implementation support is a combination of internal employees and external partners.<br>• Four internal workplace employees with partial dedication (between 4-8 hours per month) and one external consultant (allocated 127 hours for the period of the pilot).<br>• Costs include staff-time and salary dedicated to management, oversight and support of the testing program and the program manager.
Staff: Service delivery	• 1 person was hired to be a dedicated full-time program manager of the testing program for the duration of the pilot.<br>• Duties include enrolment, training, test distribution, and data collection/management.
Data and reporting	• Self-test results are reported to the program manager and uploaded to the digital platform.<br>• The cost of the digital platform comprises development costs incurred by IT personnel (80 hours multiplied by staff hourly rate).<br>• Data and reporting costs also include the cost to host meetings/working groups four times over the course of the pilot for 7 people on average at a cost of $57 per person per meeting and $150 per meeting (venue, catering).<br>• Staff time costs that relate activities conducted for data and reporting are included under the staff cost categories.
Consumables and supplies	• Not applicable for this pilot, as testing is performed off-site.
Other	• The pilot was conducted at one site. The pilot took place from September 2022 to February 2023. Costing and data collection was completed in December 2022; therefore, some costs and test results were extrapolated for the remaining 2 months of the pilot.

For peer review only - http://bmjopen.bmj.com/site/about/guidelines.xhtml

Table A2. Cost categories with descriptions for Afogados da Ingazeira, Brazil. For every cost category, all costs included, and any assumptions made are listed. All costs are reported in 2022 USD.

Cost category	Description & explanation
Test procurement	• COVID-19 self-test kit: Alerta COVID-19 Ag Self-Test manufactured by WAMA Diagnóstica based in Sao Carlos, Brazil<br>• Self-test unit price (including shipping, taxes, duties, custom clearance): $2.00<br>• 26,000 self-tests procured
Test distribution	• Tests were moved from a central storage facility to the Education and Health Secretariat offices using a municipal transport system at no extra cost.<br>• Tests were distributed from Education and Health Secretariat offices to the respective managers of each of the 34 sites included in the pilot study. During the time of the pilot, fuel costs were observed to be $1/liter. Considering the fuel economy for a light duty vehicle in Brazil and maintenance expenses, transportation of 26,000 self-tests to all 34 sites was calculated to be $850.
Test storage	• Tests were stored at an existing storage facility that was centrally located to the Education and Health Secretariats where they were delivered by the manufacturer. No additional cost was incurred from use of the storage space.<br>• 4 palletes to place the boxes holding self-tests were purchased at $37.10 each.
Training	• 2 training workshops were hosted and catered for, with a session providing instructions on how to use the self-test. $400 was budgeted for each training workshop.<br>• A training guide was provided to pilot staff to sensitise staff regarding different self-test procedures as courtesy of the test manufacturer (no cost associated).<br>• 12 hours of initial training hosted by an external consulting agency for a fixed once-off cost of $1656.<br>• Subsequent training sessions on self-test distribution and use were hosted online at no extra cost.
Communication	• Three review meetings were hosted and catered to discuss and communicate the launch of the pilot, progress, and results of the self-test pilot. $400 was budgeted for each review meeting.<br>• Advertisement posters, pamphlets, and stickers were designed and distributed to the beneficiaries at initiation. The communication materials explain when to use tests and what to do depending on the result. The materials cost $220.
Staff: Management	• Project implementation support is provided by two external consulting agencies at a cost of $552 USD per month, as well as a fixed once-off cost of $7,112.
Staff: Service delivery	• 34 individuals comprising four different working groups (operational team, monitoring team, quality control team and management team) contributed approximately 5% of their working time to the project at a cost of $24.29 per individual per month.
Data and reporting	• Self-tests were recorded by beneficiaries in a Google Form that was sent out weekly. No cost was associated with the distribution and design of the Google Form.<br>• Data was managed by two site managers at a rate of $30 per individual per month.
Consumables and supplies	• Not applicable for this pilot, as testing is performed off-site.
Other	• The pilot was conducted at a total of 34 sites. The pilot took place during October 2022 to March 2023. Costing and data collection was completed in December 2022; therefore, some costs and test results were extrapolated for the remaining 3 months of the pilot.

For peer review only

Table A3. Cost categories with descriptions for Georgia. For every cost category, all costs included, and any assumptions made are listed. All costs are reported in 2022 USD.

Cost category	Description & explanation
Test procurement	● COVID-19 self-test kit: On-site COVID-19 Ag Self-Test® (CTK Biotech, California, USA)<br>● Self-test unit price (including shipping, taxes, duties): $2.00<br>● 90,0000 self-tests procured
Test distribution	● Fuel for pilot staff to transport tests from the National Centres for Disease Control (NCDC) to Primary Healthcare Center in Mestia, and from there to schools in the Svaneti region.<br>● Only costs for transportation of tests to schools are accounted for. The NCDC already makes regular trips to the health care facilities and nursing homes for delivery of other supplies through an established delivery system. The additional cost, as well as the opportunity cost of distributing self-tests was considered negligible.
Test storage	● No additional storage space had to be rented to store tests. Every site owned a storage of their own where tests were stored and there was no opportunity cost to the storing of tests.<br>● No additional supplies were purchased to store tests.<br>● Storage of the tests at NCDC was considered negligible.
Training	● A workshop for each of the modalities was provided to train staff (facilitators, principal investigators, project managers) to perform actions needed in the pilot. Costs for these workshops consisted of fuel for transportation, flights (for the school modality), printing supporting materials, supplies (hand sanitizers and masks), venue and hotel costs. - a total of 5 trainings<br>● Allocation of shared training costs for schools, nursing homes and hospital/clinic modalities was based on the number of facilitators allocated to the different modalities.
Communication	● NCDC Communication staff (marketer, social media manager, designer, health promotion, social media coordinator).<br>● Making of videos, social media posts etc. on self-testing and general testing.<br>● Pens, bags, brochures, posters, banners.
Staff: Management	● Salary cost of time spent by:<br>○ Principal investigators (PI and co-PI) at NCDC<br>○ Project managers (PM) at NCDC as well as sites (total of 15)<br>○ Project implementation and support including consultant time<br>○ Finance and admin support from NCDC<br>● Used average Georgian salaries for the current staff position (instead of a stipend paid to staff by FIND to conduct the pilot) to ensure better alignment with the actual costs of scaling up test distribution.<br>● Estimated the average proportion of their time each month that is dedicated to COVID-19 self-test implementation and operations.<br>● Pilot-related staff, such as PMs included as time largely spent on implementation (with a small component spent on research-specific activities).<br>● Included 2 months preparation time for the pilot.
Staff: service delivery	● This includes the salary of the facilitators at each of the sites (NCDC facilitators (9), hospitals (24), clinics (7), nursing homes (4), and schools (24), and then the average proportion of their time each month that is dedicated to COVID-19 self-test operations.<br>● Facilitator activities included:<br>○ Facilitator time spent on collecting COVID-19 self-tests to bring to appropriate testing facility (i.e., workplace, schools)<br>○ Facilitator time spent on storing and organizing COVID-19 self-tests<br>○ Facilitator time spent on distributing the COVID-19 self-test to participants<br>○ Facilitator time spent on training clients/participants on how to use the COVID-19 self-test, including information and sensitization activities

For peer review only - http://bmjopen.bmj.com/site/about/guidelines.xhtml

	• Used average Georgian salaries for the current staff position (instead of a stipend paid to staff by FIND to conduct the pilot) to ensure better alignment with the actual costs of scaling up test distribution.
Data and reporting	• Costs associated with the development and use of a database/app where results are documented and reported.<br>• Staff time of data entry operators at hospitals (6), clinics (2) and nursing homes (2). (Facilitators conduct data entry at schools). Data entry operators are responsible for record keeping after COVID-19 self-tests are distributed, maintaining registers, data compilation and entry. Beneficiaries test weekly and, if positive, call in immediately for the data operator to record it. If negative, the beneficiary will submit the result at work.<br>• Technical Working Group meetings to report and share results (venue, food, IT, and transportation) – a total of 3.<br>• Data support from the NCDC in terms of staff time (5).

For peer review only

For peer review only - http://bmjopen.bmj.com/site/about/guidelines.xhtml

Table A4. Cost categories with descriptions for Malaysia. For every cost category, all costs included, and any assumptions made are listed. All costs are reported in 2022 USD.

Cost category	Description & explanation
Test procurement	• COVID-19 self-test kit: FlowFlex Ag-RDTs manufactured by ACON Biotech (Hangzhou) Co., Ltd.<br>• Self-test unit price (including shipping, taxes, duties, custom clearance): $1.55<br>• 45,000 self-tests procured
Test distribution	• Tests were shipped from the central storage facility to each site through a third-party courier at a rate of $0.0121 per test. This cost included door-to-door delivery, driver salary, fuel, vehicle, etc.<br>• For the 45,000 tests procured for the pilot, test distribution costs totalled $545.
Test storage	• Tests were stored in a central storage facility in Kuala Lumpur at a cost of $420 per month.<br>• A temperature data logger was purchased to ensure temperature regulation of the storage facility as a once off cost of $20.
Training	• 2 in-person training sessions were hosted for a research assistant dedicated to the self-testing pilot. Travel associated with attending each session totalled $286.<br>• 1 training session was hosted at each site (4 total) to train site managers on the self-testing pilot. $915 was incurred for each training session per site.<br>• A sensitization session was hosted at each site (4 total) to train beneficiaries on how to use the FlowFlex Ag-RDT. $458 was incurred for each training session per site.
Communication	• Supplementary information and reference guides for the self-tests were provided to each beneficiary. $1030 was budgeted for these communication materials.<br>• 3 working group meetings were hosted to monitor and communicate upon the reception and progress of the COVID-19 self-testing pilot. $1145 was budgeted for each working group meeting.
Staff: Management	• Project implementation support is provided by an external organisation for $1438 per month.
Staff: Service delivery	• A research assistant was dedicated to delivering the self-tests to their respective sites and hosting the sensitization sessions for the beneficiaries. Salary and travel expenses for this position resulted in a total cost of $1145 per month.
Data and reporting	• Self-tests were recorded by beneficiaries in a Google Form that was sent out weekly and managed by the project's dedicated research assistant. No additional cost was associated with the distribution and management of the Google Form.<br>• A sim card was purchased to follow-up with beneficiaries via phone who did not report their test results on the Google form for $11 per month.
Consumables and supplies	• Not applicable for this pilot, as testing is performed off-site.
Other	• The pilot was conducted at a total of 2 sites. The pilot took place during November 2022 to April 2023. Costing and data collection was completed in December 2022; therefore, some costs and test results were extrapolated for the remaining 2 months of the pilot.

For peer review only - http://bmjopen.bmj.com/site/about/guidelines.xhtml

Table A5. Cost categories with descriptions for Ethiopia. For every cost category, all costs included, and any assumptions made are listed. All costs are reported in 2022 USD.

**Cost category**	**Description & explanation**
Test procurement	● COVID-19 self-test kit: Flowflex Acon Biotech self-test kit, ACON Biotech (Hangzhou) Co.,Ltd.<br>● Self-test unit price: $1.00<br>● 1,250 self-tests procured
Test distribution	● Transportation cost from the airport to health care facilities. Once-off cost ($140) for all imported tests used to estimate a cost per self-test kit transported.
Test storage	● No additional storage space had to be rented to store tests. Every site owned a storage of their own where tests were stored and there was no opportunity cost to the storing of tests.<br>● No additional supplies were purchased to store tests.
Training	● A video was produced and procured for self-test kit demonstration purposes. Field coordinators initially watched the video themselves (as part of training). The video was also shown to patients to demonstrate proper self-test kit usage.<br>● Once-off cost of $500 for video production.
Communication	● Mobile card usage for four field coordinators (2 per site) to provide updates on the study.<br>● Monthly cost per mobile card: $14.81
Staff: Management	● Salary cost of time spent by:<br>○ Cleaner, administrative assistant, monitoring & evaluation officer, and program coordinator at sites (total of 8 sites)<br>○ Project implementation and support including consultant time from implementing partner<br>○ Finance and administration support from implementing partner<br>● Used average Ethiopian salaries for the current staff position (instead of a salary paid by the implementing partner to conduct the pilot) to ensure better alignment with the actual costs of scaling up test distribution.<br>● Estimated the average proportion of their time each month that is dedicated to COVID-19 self-test implementation and operations.<br>● Allocated the time of all staff to the 2 sites used in the costing analysis based on test-kit distribution For the routine implementation scenario analysis, the level of effort of the monitoring and evaluation officer was reduced, and the salary cost of the monitoring and evaluation officer and programme coordinator was aligned with that of the staff cadre that would likely fill these positions should the programme be scaled.
Staff: Service delivery	● This includes the salary of the OPD physicians (1 per site), nurses and field coordinators (2 per site) at each of the sites (2 sites).<br>● Staff: service delivery activities included:<br>○ OPD physicians: time spent on evaluation clients if they are eligible for self-testing and linking them to field coordinators (1 hour per day)<br>○ Nurses: time spent on observing clients execute self-testing, collect, and process the sample and finally read the result<br>○ Field coordinators: time spent on arrival and registration of client, showing client a demonstration video, pre-test counselling, preparing the testing area and conclude by providing post-test counselling/information<br>● The direct staff cost for nurses and field coordinators was estimated on a per test basis by observing time estimates spent on service delivery and salary cost per minute: staff nurse (25 minutes per client); field coordinator (42 minutes per client).

For peer review only - http://bmjopen.bmj.com/site/about/guidelines.xhtml

	• Used average Ethiopian salaries for the staff cadre required to perform the task (instead of a salary paid to staff by the implementing partner to conduct the pilot) to ensure better alignment with the actual costs of scaling up test distribution.
Data and reporting	• Field coordinators provide post-counselling/information to clients and document and report the self-test result.<br>• Unlimited internet SIM card procured for four field coordinators (acting as data collectors; 2 per site) for facilitation of data entry on REDCap: $16.67 per month.
Consumables and supplies	• Costed hand sanitizer, gloves, and alcohol for disinfecting the testing area on a per test basis.<br>• Costed masks and biohazard bags used by the facility and staff to oversee the self-testing.
Other	• The pilot was conducted at a total of 8 sites. However, given costing timelines, costs were only collected for 2 sites. These sites are however representative of the other sites.<br>• Costs for the video production and overhead staff management were allocated to the 2 sites based on test-kit distribution (46% of test kits were distributed at the 2 sites that were costed).

For peer review only

Table A6. Cost categories with descriptions for the Philippines. For every cost category, all costs included, and any assumptions made are listed. All costs are reported in 2022 USD.

Cost category	Description & explanation
Test procurement	• COVID-19 self-test kit: Flowflex Acon Biotech self-test kit, ACON Biotech (Hangzhou) Co.,Ltd.<br>• Self-test unit price: $1.32 (including shipment of test from the local distributor – taxes and duties)<br>• 2,000 self-tests procured
Test distribution	• Transport billed in bulk inclusive of porter's/driver's salary, vehicle rental, and applicable fuel, toll fees, and taxes. Fixed cost per day, per site, per month.<br>• Tests are distributed directly to the partner sites.
Test storage	• No additional storage space had to be rented to store tests at the central office. Store to capacity and re-order when need arises.<br>• Each site has storage of its own and no opportunity cost to the storing of tests.
Training	• A 1-day training workshop was conducted with all partner site staff (50 in total).<br>• Each participant received 2 demonstration tests.<br>• A cost per person trained per day was calculated to estimate the cost of training the staff at one site.
Communication	• All site staff receive a monthly communication allowance inclusive of phone, internet service provider subsidy and data transfer to servers.<br>• Management staff also receive a monthly communication allowance to manage all 7 sites. This cost is allocated to the costed site using 1/7 factor.<br>• Printing papers, stationery, cartridges for brochures, Information Education Communication (IEC) materials, and posters, banners, and brochures – once-off per site.
Staff: Management	• 1 x Project implementer Research officer (supporting all sites).<br>• 1x Project M&E Officer – solely contracted by project implementer to supervise across all sites.<br>• 1 x Site Supervisor (clinic staff) at nurse salary grade 19 (government salary grade)<br>• Estimated the average proportion of their time each month that is dedicated to COVID-19 self-test implementation and operations:<br>○ 5 hours per 8-hour day for the Project implementer Research officer<br>○ 5 hours per 8-hour day for the Project M&E Research officer<br>○ 1 hour per 8-hour day for the Site Supervisor (clinic staff)<br>• Research staff time spent on self-test distribution allocated per site (monthly salary/7).<br>• M&E Research staff time spent on self-test distribution allocated per site (monthly salary/7).<br>• Included 1-month of preparation time.
Staff: Service delivery	• 1x Test counsellor –at medical technologist II salary grade 15 (government salary grade).<br>• 1x Test administrator – at Nurse Salary I with salary grade 15.<br>• Estimated the average proportion of their time each month that is dedicated to COVID-19 self-test implementation and operations (3 hours per 8-hour day).
Data and Reporting	• 1x Site Data Entry Operator – Project implementer assigned staff on-site, Nursing Attendant II w/ Salary Grade 6 (government salary grade).<br>• Estimated the average proportion of their time each month that is dedicated to COVID-19 self-test implementation and operations:<br>○ 100% of their time for the Data Entry Operator

For peer review only - http://bmjopen.bmj.com/site/about/guidelines.xhtml

Consumables and Supplies	• Hygiene essentials: infection control kits available on-site as part of IPC procedures (alcohol, masks, gloves, hand soap, paracetamol, disinfectants, thermometer).<br>• Office supplies for staff: documentation and office essentials (printing papers, stationery, cartridges, ball pens).
Other	• The pilot was conducted at a total of 7 sites. Only 1 site was costed. This site was considered typical of the other sites; set-up is the same across sites, only the scale of testing differs.<br>• Costs for the staff that oversee operations across sites, their communication allowance and the supplies and consumables were allocated to one site based on the factor of 1/7.

For peer review only

Table A7. Program characteristics and all-inclusive cost per test. These characteristics and costs are reported for each country during the pilot (Scenario 1) and annual routine periods of implementation (Scenario 2 and 3). This is an extended version of Table 4 in the manuscript.

			**Scenario 1 – pilot implementation**			**Scenario 2 – routine implementation**			**Scenario 3 – scaled peak implementation**			
**Country**	**Testing location**	**Provider assisted or unassisted**	**# tests distributed**	Purchase price of self-test kit	**Cost per test:** all-inclusive	**# tests distributed**	**Cost per test:** using current self-test kit price	**Cost per test:** using reduced self-test kit price	**# tests distributed**	**Cost per test:** using current self-test kit price	**Cost per test:** using reduced self-test kit price	**Cost per test:** with integrated staff, data, & reporting
**Brazil (Pelotas)**	Primarily off-site	Unassisted	**10,000**	$4.50	$7.38	**20,000**	$6.54	$4.29	**40,000**	$5.62	$3.37	$2.55
**Brazil (Afogados)**	Primarily off-site	Unassisted	**26,000**	$2.00	$2.82	**52,000**	$2.59	$1.59	**104,000**	$2.30	$1.30	$1.05
**Georgia**	Off-site	Unassisted	**90,000**	$2.00	$4.44	**300,000**	$3.34	$2.34	**600,000**	$2.95	$1.95	$1.61
**Malaysia**	Off-site	Unassisted	**45,000**	$1.55	$2.44	**135,000**	$2.06	$1.28	**270,000**	$1.82	$1.04	$0.86
**Ethiopia**	On-site	Provider assisted	**158**	$1.00	$12.78	**5,040**	$4.22	$3.72	**10,080**	$3.80	$3.30	$1.97
**Philippines**	On-Site	Unassisted	**2,000**	$1.32	$4.99	**6,000**	$4.51	$3.85	**12,000**	$2.92	$2.26	$0.95

For peer review only - http://bmjopen.bmj.com/site/about/guidelines.xhtml

Table A8. CHEERS Checklist

Topic	No.	Item	Location where item is reported
**Title**			
	1	Identify the study as an economic evaluation and specify the interventions being compared.	Title
**Abstract**			
	2	Provide a structured summary that highlights context, key methods, results, and alternative analyses.	Abstract
**Introduction**			
**Background and objectives**	3	Give the context for the study, the study question, and its practical relevance for decision making in policy or practice.	Introduction, starting at line 74
**Methods**			
**Health economic analysis plan**	4	Indicate whether a health economic analysis plan was developed and where available.	Methods, starting at line 129 and 147
**Study population**	5	Describe characteristics of the study population (such as age range, demographics, socioeconomic, or clinical characteristics).	Methods, start at line 129
**Setting and location**	6	Provide relevant contextual information that may influence findings.	Methods, start at line 140
**Comparators**	7	Describe the interventions or strategies being compared and why chosen.	Methods, start at line 172
**Perspective**	8	State the perspective(s) adopted by the study and why chosen.	Methods, line 148
**Time horizon**	9	State the time horizon for the study and why appropriate.	Methods, line 151
**Discount rate**	10	Report the discount rate(s) and reason chosen.	Not applicable
**Selection of outcomes**	11	Describe what outcomes were used as the measure(s) of benefit(s) and harm(s).	Not applicable
**Measurement of outcomes**	12	Describe how outcomes used to capture benefit(s) and harm(s) were measured.	Not applicable
**Valuation of outcomes**	13	Describe the population and methods used to measure and value outcomes.	Not applicable
**Measurement and valuation of resources and costs**	14	Describe how costs were valued.	Methods, line 147
**Currency, price date, and conversion**	15	Report the dates of the estimated resource quantities and unit costs, plus the currency and year of conversion.	Methods, line 150

For peer review only - http://bmjopen.bmj.com/site/about/guidelines.xhtml

Topic	No.	Item	Location where item is reported
**Rationale and description of model**	16	If modelling is used, describe in detail and why used. Report if the model is publicly available and where it can be accessed.	Not applicable
**Analytics and assumptions**	17	Describe any methods for analysing or statistically transforming data, any extrapolation methods, and approaches for validating any model used.	Methods, line 172
**Characterising heterogeneity**	18	Describe any methods used for estimating how the results of the study vary for subgroups.	Methods, starting at line 172
**Characterising distributional effects**	19	Describe how impacts are distributed across different individuals or adjustments made to reflect priority populations.	Methods, starting at line 172
**Characterising uncertainty**	20	Describe methods to characterise any sources of uncertainty in the analysis.	Methods, starting at line 172
**Approach to engagement with patients and others affected by the study**	21	Describe any approaches to engage patients or service recipients, the general public, communities, or stakeholders (such as clinicians or payers) in the design of the study.	See appendices
**Results**			
**Study parameters**	22	Report all analytic inputs (such as values, ranges, references) including uncertainty or distributional assumptions.	Not applicable
**Summary of main results**	23	Report the mean values for the main categories of costs and outcomes of interest and summarise them in the most appropriate overall measure.	Results, Figure 1
**Effect of uncertainty**	24	Describe how uncertainty about analytic judgments, inputs, or projections affect findings. Report the effect of choice of discount rate and time horizon, if applicable.	Not applicable
**Effect of engagement with patients and others affected by the study**	25	Report on any difference patient/service recipient, general public, community, or stakeholder involvement made to the approach or findings of the study	Results, starting at line 212
**Discussion**			
**Study findings, limitations, generalisability, and current knowledge**	26	Report key findings, limitations, ethical or equity considerations not captured, and how these could affect patients, policy, or practice.	Discussion, starting at line 254
**Other relevant information**			
**Source of funding**	27	Describe how the study was funded and any role of the funder in the identification, design, conduct, and reporting of the analysis	Line 442
**Conflicts of interest**	28	Report authors conflicts of interest according to journal or International Committee of Medical Journal Editors requirements.	Line 437

For peer review only - http://bmjopen.bmj.com/site/about/guidelines.xhtml
